# Bile acids enrichment fuel tumor aerobic glycolysis and immune evasion via stabilizing FXR-RARα

**DOI:** 10.3389/fimmu.2026.1750358

**Published:** 2026-03-25

**Authors:** Hongyu Jiang, Wenyan Xiong, Yingna Feng, Xingjie Li, Jinzhuo Tan, Zongde Zhang

**Affiliations:** 1 Inflammation and Allergic Diseases Research Unit, The Affiliated Hospital, Southwest Medical University, Luzhou, Sichuan, China; 2 School of Basic Medical Sciences, Southwest Medical University, Luzhou, Sichuan, China

**Keywords:** aerobic glycolysis, bile acids (BAs), farnesoid X receptor (FXR), immune evasion, retinoic acid receptor alpha (RARα), tumor microenvironment (TME)

## Abstract

**Introduction:**

Fast-growing solid tumors exhibit aerobic glycolysis to meet metabolic demands and evade immune surveillance. While the tumor microenvironment (TME) plays a crucial role in supporting this glycolytic phenotype, the contribution of host metabolites remains incompletely understood.

**Methods:**

The role of bile acids (BAs) in tumor progression was evaluated using multiple mouse models, including C57BL/6J mice injected with B16 melanoma cells, BALB/c mice inoculated with 4T1 breast cancer cells, and MMTV-PyMT mice with spontaneous tumor development. To assess the impact of BA depletion, subjects were fed a 2% cholestyramine diet compared to a regular diet. The study employed various analytical techniques, including UPLC-MS to evaluate BA profiles, flow cytometry for immune cell analysis, and Seahorse extracellular flux analyzers to measure oxygen consumption and extracellular acidification rates.

**Results:**

Here, we demonstrate that multiple bile acids (BAs) are significantly enriched in the TMEs of melanoma and breast cancer. Mechanistically, this BA enrichment drives tumor aerobic glycolysis and promotes immune evasion by modulating the interaction between the farnesoid X receptor (FXR) and retinoic acid receptor alpha (RARa). Depletion of BAs in vivo suppressed tumor progression, enhanced T cell infiltration, and alleviated T cell exhaustion, accompanied by transcriptome-wide shifts towardincreased expression of genes involved in oxidative phosphorylation. Furtherinvestigations revealed that BAs activate and stabilize the FXR-RARa complex,thereby upregulating glycolytic pathways and impairing anti-tumor immunity. Conversely, BA depletion reduced FXR/RARa protein levels and disrupted tumorimmune barrier function.

**Discussion:**

These findings unveil a mechanism by which bile acids promote tumor progression by modulating tumor metabolism and the immune microenvironment through FXR-RARa signaling. Targeting the BA-FXR-RARa axiscould offer promising strategies for cancer therapy and diagnosis.

## Introduction

Tumor cells primarily depend on aerobic glycolysis for energy production, a metabolic shift known as the Warburg effect, which is considered a hallmark of cancer malignancy (1). Beyond providing ATP for cellular energy, aerobic glycolysis is increasingly recognized as a key adaptation for tumor cells, facilitating biosynthetic pathways and contributing to a tumor microenvironment that supports proliferation and suppresses anti-tumor immunity. This process generates essential metabolic intermediates utilized in the biosynthesis of macromolecules to support cell proliferation (2, 3) and modulates tumor progression by producing lactate, functioning as a proinflammatory and immunosuppressive mediator (1, 4). Increased tumor aerobic glycolysis also promotes immune resistance, including resistance to adoptive T-cell therapy (5) and immune evasion by reducing sensitivity to T-cell-mediated killing (6). While genetic alterations in oncogenes and tumor suppressors, such as TP53 and KRAS, can stimulate aerobic glycolysis (7), these genetic drivers alone are insufficient to explain the pervasive nature of this metabolic reprogramming across diverse cancers. Non-cell-autonomous factors within the tumor microenvironment may play a critical role in shaping tumor metabolism. Understanding these factors is thus paramount for developing novel therapeutic approaches to target cancer metabolism, potentiate immunotherapy responses, and overcome resistance.

Among the diverse components of the TME, metabolites originating from the host and gut microbiota are increasingly recognized as crucial regulators of tumor biology. Bile acids (BAs), bioactive molecules produced by the host and extensively modified by gut bacteria (8), have recently emerged as intriguing candidates for mediating TME influence on tumor metabolism. Primary BAs are synthesized in the liver and conjugated to glycine or taurine before secretion into the duodenum. Most primary BAs are stored in the gallbladder; the microbiota converts the others to secondary BAs in the intestinal tract (8). BAs are associated with gastrointestinal and hepatobiliary cancers (9). However, the mechanism by which bile acids influence tumor growth remains unclear.

There are two central BAs sensing receptors: farnesoid X receptor (FXR) and G protein-coupled bile acid receptor (TGR5) (10). FXR is a responsive receptor that could regulate tumor epithelial cells (9, 11). Solid tumor tissues producing retinoic acid induce immunosuppression in the tumor microenvironment (12, 13). The retinoic acid receptor α (RARα) is a major transcriptional factor, and its fusion with the PML gene forms the well-known oncogenic driver PML-RARα, which promotes acute promyelocytic leukemia (APL) (14). Whether bile acid could stimulate FXR-RARα in tumor cells needs to be determined.

Here, we report that bile acids were enriched in TME with transplanted melanoma and spontaneous murine breast tumor models. Bile acids enrichment sustains tumor aerobic glycolysis by activating and stabilizing the FXR-RARα complex. Bile acid depletion impairs tumor progression, promotes lymphocyte infiltration, and stimulates an anti-tumor immune response.

## Results

### Solid tumors are enriched in bile acids and rely on them to sustain tumor growth

**Figures 1:**
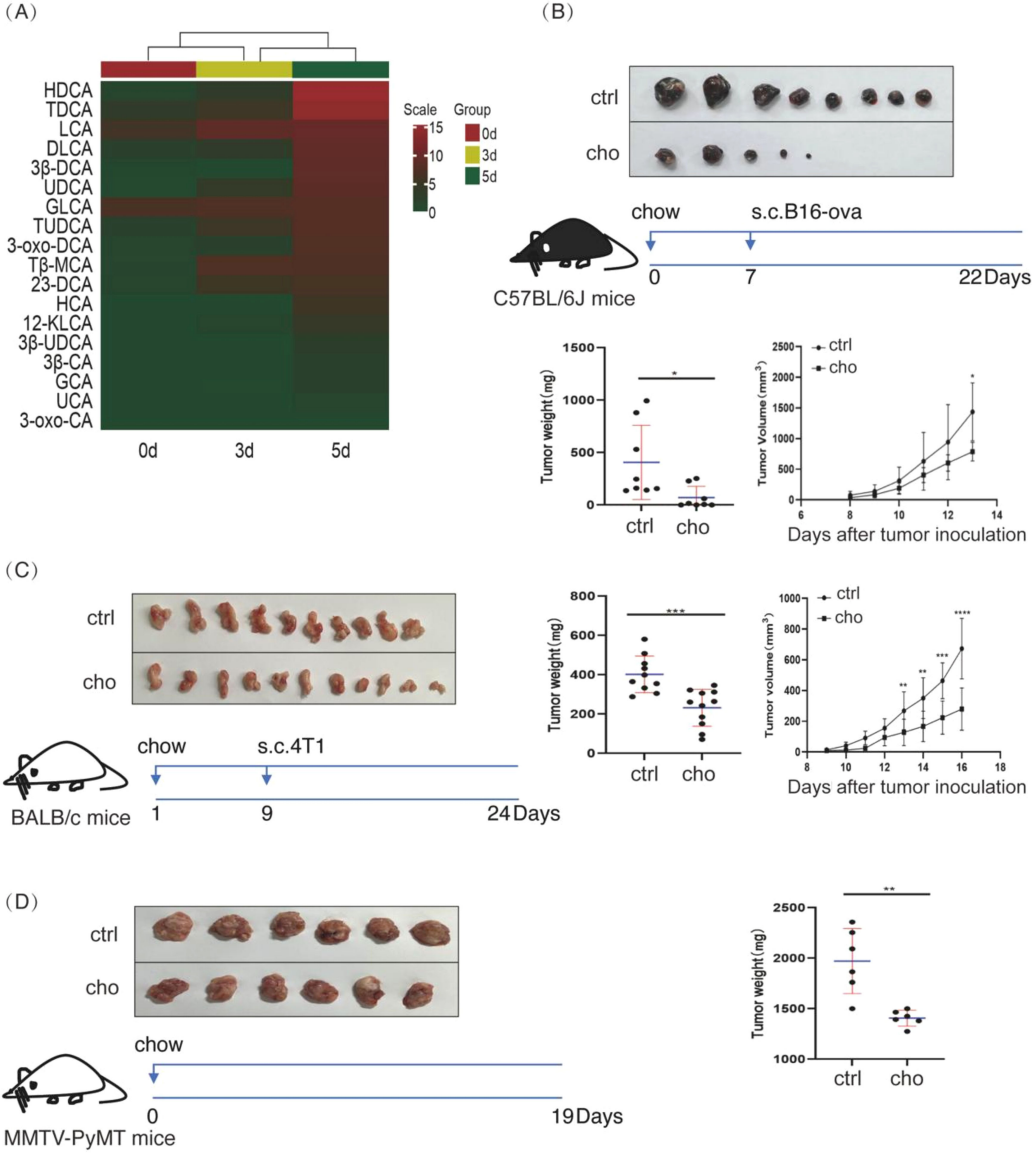
Depleting bile acids inhibits tumor growth in multiple mouse models. **(A)** C57BL/6J mice were subcutaneously injected with 0.5 × 10^^6^ B16 cells on the left lower back. On the 0th, 3rd, and fifth days, UPLC-MS analysis of bile acid profiles in melanomas was performed. The hierarchical clustering datasets were processed in R. **(B)** Wild-type C57BL/6J mice (5–6 weeks, male, n=8) were fed 2% cholestyramine or a regular diet. A week later, mice were injected subcutaneously with 0.5 × 10^^6^ B16-OVA cells on the left lower back. Half a month later, mice were sacrificed. Representative melanoma images are shown. **(C)** BALB/c mice (4–5 weeks, female, control=10, cholestyramine=11) were fed 2% cholestyramine or a regular diet. Eight days later, mice were given a subcutaneous injection of 2.5 × 10^4 T1 cells in the left lower back. Half a month later, mice were sacrificed. Representative tumor images are shown. **(D)** MMTV-PyMT mice (5–6 weeks, female, n=6) were fed 2% cholestyramine or a regular diet. Nineteen days later, mice were sacrificed. Representative tumor images are shown. Data are presented as mean ± SD; p-values were calculated using the two-tailed Student’s t-test. *p < 0.05, **p < 0.01, ***p < 0.001, ****p < 0.0001, n.s., not significant.

First, we determine whether bile acid profiles in tumor tissue change during tumor growth. B16 melanoma cells were transplanted into C57BL/6J mice. Tumor tissues were collected at 0, 3, and 5 days, and UPLC-MS was used to analyze the bile acid profiles. Our analysis revealed a progressive increase in bile acid concentration during tumor growth (**Figure 1A**). Specifically, Hyodeoxycholic acid (HDCA), Taurodeoxycholic acid (TDCA), and Lithocholic acid (LCA) showed significantly elevated levels in tumor tissue compared to surrounding normal tissue (**Figure 1A**). Next, we reasoned that bile acid enrichment might sustain B16 melanoma cells’ growth. To test this conjecture, we fed mice a cholestyramine-containing diet to deplete bile acids and assess the growth of transplanted B16 melanoma cells. Notably, cholestyramine treatment significantly reduced tumor mass (**Figure 1B**). In several cholestyramine-treated mice, the melanoma tumors were undetectable. To assess the generalizability of this finding, we replicated the experiment in a 4T1 murine breast cancer transplant model in BALB/c mice. Consistently, depletion of bile acids with cholestyramine robustly inhibited 4T1 breast cancer growth in BALB/c mice (**Figure 1C**). Furthermore, we investigated whether bile acid depletion could impact spontaneous tumor development using MMTV-PyMT transgenic mice, which spontaneously develop breast cancer. MMTV-PyMT mice fed a cholestyramine diet significantly reduced tumor volume and weight (**Figure 1D**), indicating that bile acid depletion impedes spontaneous tumor growth. Next, we examined the expression of bile acid transporters in transplanted B16 melanoma. As shown (**Supplementary Figure 6**), these bile acid transporters (OATP1, OATP4, IBABP, OSTb, ASBT (SLC10A2), NTCP) are highly expressed in melanoma xenografts (0d), but decreased after tumor expansion (3d, 5d), which may suggest a feedback inhibition by bile acid enrichment. These collective *in vivo* data strongly suggest that bile acid enrichment is not merely a correlation but actively contributes to sustaining and promoting solid tumor growth.

### Bile acid enrichment promotes tumor aerobic glycolysis

**Figure 2:**
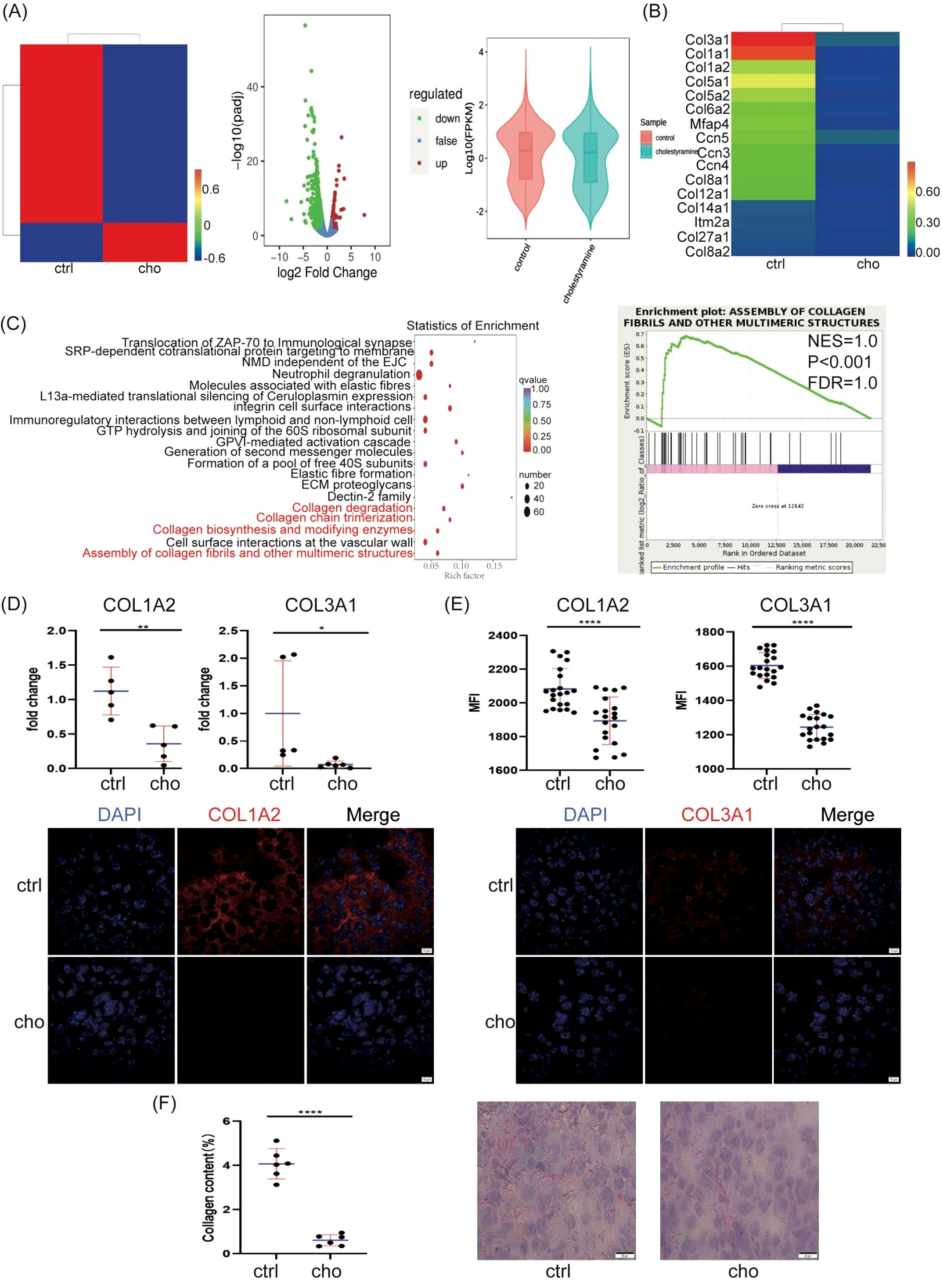
The effect of depleting bile acid on collagen in a melanoma model. **(A)** RNA seq analysis of global gene expression of control and cholestyramine (n=3 per group, pooled together) in the C57BL/6J mouse melanoma model. The heatmap shows fragments per kilobase per million reads; volcano plots show log10(padj) *vs*. log2(fold change); and a boxplot of the FPKM density distribution is shown. **(B)** Genes related to collagen biosynthesis, modification, and assembly in RNA seq. **(C)** RNA seq analysis of collagen fibrils enrichment plot. The profiles of the running ES score and positions of gene set members, as well as gene set enrichment plots from GSEA (NES-Normalized enrichment score; FDR-False discovery rate; P-Family-wise error rate). **(D)** RT-qPCR determined COL1A2 mRNA and COL3A1 mRNA expression levels in melanomas. **(E)** Immunofluorescent analysis of COL1A2 and COL3A1 in melanomas by 2% cholestyramine-fed mice. Melanomas were stained with DAPI (blue), COL1A2 (red), or COL3A1 (red) antibodies and analyzed by confocal imaging. The scale bars are 10μm. **(F)** Collagen fiber in melanomas by 2% cholestyramine chow mice. Melanomas were stained with Sirius Red staining. The scale bars are 20μm. The statistical quantification was analyzed using ImageJ software. Data are presented as mean ± SD; p-values were calculated using the two-tailed Student’s t-test. *p < 0.05, **p < 0.01, ***p < 0.001, ****p < 0.0001, n.s., not significant.

Next, we sought to elucidate the tumor cell-intrinsic mechanisms underlying the pro-tumorigenic effects of bile acids, with a focus on tumor metabolism. Transcriptomic analysis of cholestyramine-treated melanoma tumors compared to controls revealed significant gene expression changes (**Figure 2A**). Notably, pathways related to collagen biosynthesis were downregulated (**Figures 2B, C**), confirmed by reduced expression of COL3A1 and COL1A2 genes by RT-qPCR and confocal imaging (**Figures 2D, E**), and decreased collagen fibers as indicated by Sirius Red staining (**Figure 2F**). These data indicate that bile acid depletion impacts the tumor extracellular matrix.

**Figure 3:**
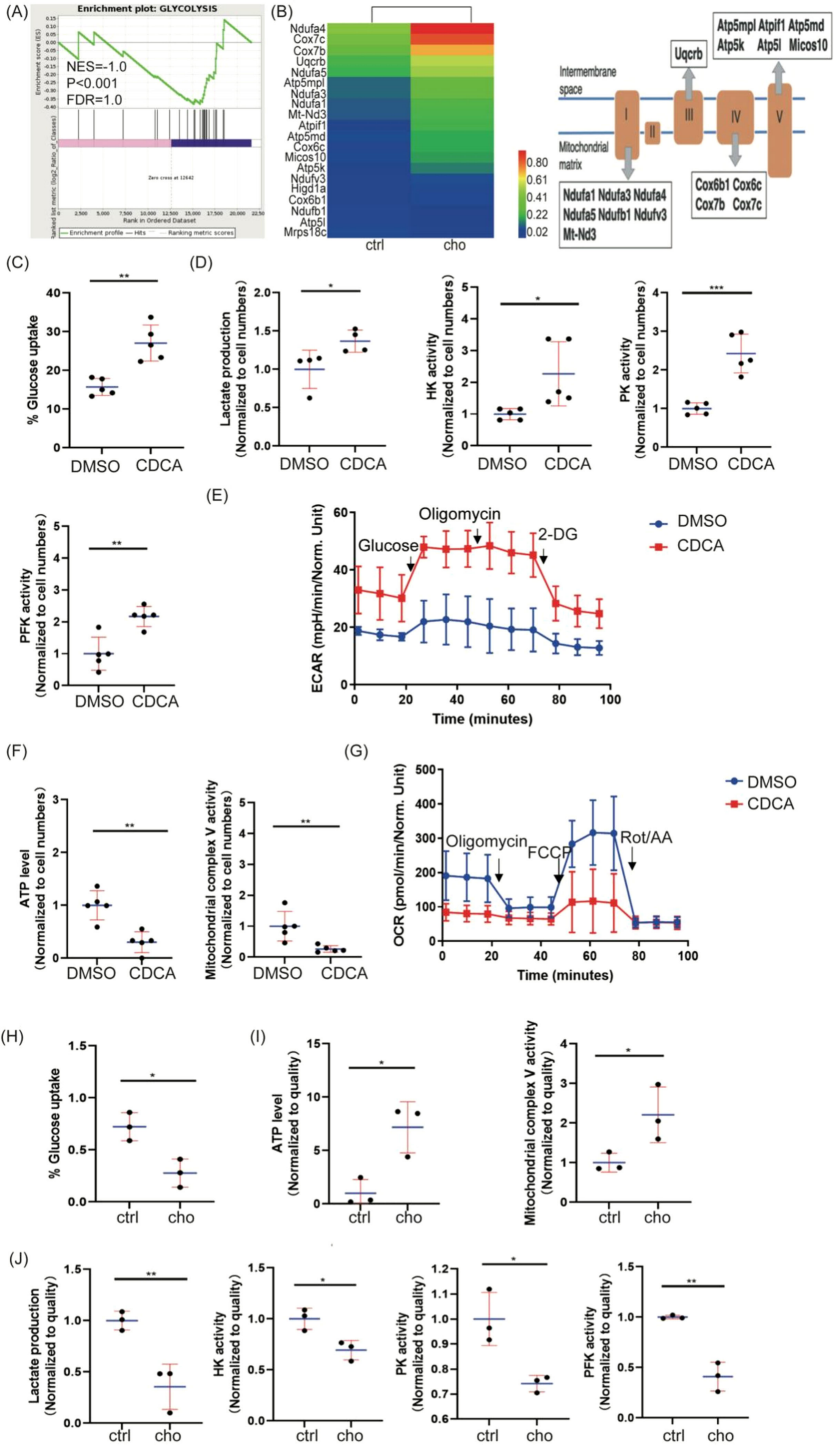
Bile acid relates to tumor aerobic glycolysis in vitro and in vivo. **(A)** RNA seq analysis glycolysis enrichment plot. Gene set enrichment plots from GSEA (NES-Normalized enrichment score; FDR-False discovery rate; P-Family-wise error rate). **(B)** Genes of the mitochondrial electron transport chain in RNA seq. **(C)** B16 cells were stimulated with CDCA and stained with 2-NBDG in a no-glucose medium, and glucose uptake was examined by flow cytometry. **(D)** B16 cells were stimulated with CDCA, and a microplate reader measured lactate production, HK activity, PK activity, and PFK activity. **(E)** B16 cells were stimulated with CDCA, and ECAR was measured using a Seahorse XFe24 Analyzer. **(F)** B16 cells were stimulated with CDCA, and ATP levels and mitochondrial complex V activity were measured using a microplate reader. **(G)** B16 cells were stimulated with CDCA, and OCR was measured using a Seahorse XFe24 Analyzer. **(H)** Melanoma cells were stained with 2-NBDG in a No-Glucose medium, and the melanoma model was examined for glucose uptake by flow cytometry. **(I, J)** The microplate reader in the melanoma model measured ATP level, mitochondrial complex V activity, lactate production, HK activity, PK activity, and PFK activity. Data are presented as mean ± SD; p-values were calculated using the two-tailed Student’s t-test. *p < 0.05, **p < 0.01, ***p < 0.001, ****p < 0.0001, n.s., not significant.

To assess the metabolic impact of bile acid depletion, we performed gene set enrichment analysis (GSEA). GSEA revealed downregulation of genes associated with glycolysis (**Figure 3A**) and concurrent upregulation of genes mapped to mitochondrial electron transport chain complexes (OXPHOC) (**Figure 3B**), suggesting that bile acid depletion shifts tumor metabolism away from glycolysis towards oxidative phosphorylation. To directly interrogate the impact of bile acids on glycolysis, we stimulated B16 cells *in vitro* with chenodeoxycholic acid (CDCA). CDCA stimulation led to increased glucose uptake, lactate production, Hexokinase (HK) activity, Pyruvate kinase (PK) activity, and Phosphofructokinase (PFK) activity in B16 cells (**Figures 3C, D**), all hallmarks of enhanced aerobic glycolysis. Moreover, extracellular acidification rate (ECAR), an indicator of glycolysis, was increased (**Figure 3E**). In contrast, oxygen consumption rate (OCR), reflecting mitochondrial respiration, was decreased in BA-stimulated B16 cells (**Figure 3G**), accompanied by decreased ATP levels and mitochondrial complex V activity (**Figure 3F**). Importantly, we validated these *in vitro* findings *in vivo* using transplanted melanoma models, observing consistent results (**Figures 3H, I**). These data conclusively demonstrate that bile acid enrichment promotes tumor aerobic glycolysis *in vitro* and *in vivo*.

### Depletion of bile acids stimulates an anti-tumor immune response

**Figure 4:**
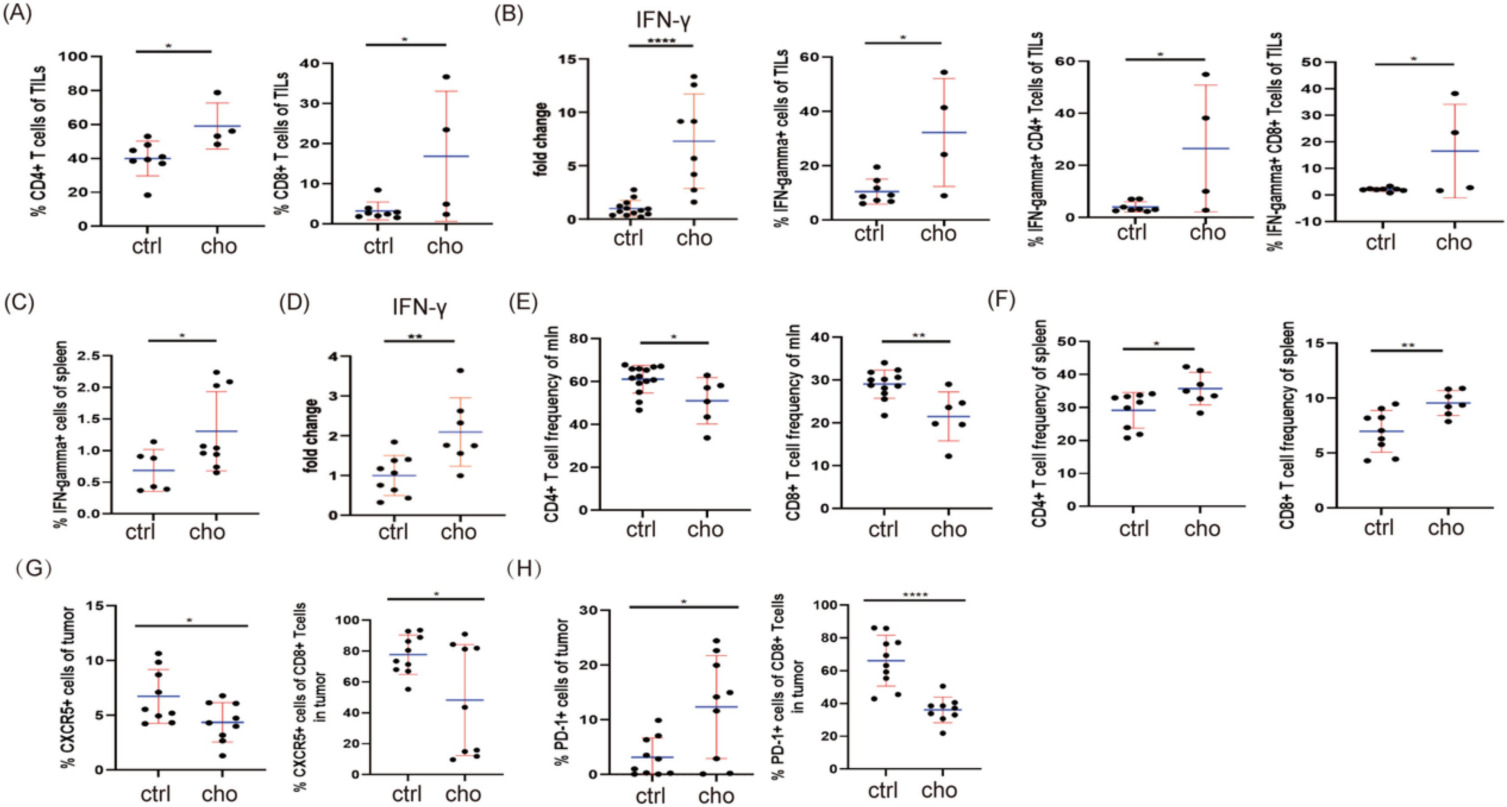
Depletion of bile acids stimulates an anti-tumor immune response. **(A)** The frequency of CD4+T cells and CD8+ T cells in TILs was analyzed by flow cytometry. **(B)** RT-qPCR analyzed IFN-γ mRNA expression in melanomas; IFN-γ+cells (CD4^+^, CD8^+^), IFN-γ^+^ cells frequency in TILs was analyzed by flow cytometry. **(C)** IFN-γ^+^ cell frequency in the spleen was analyzed by flow cytometry. **(D)** IFN-γ mRNA level was analyzed by RT-qPCR in dLN. **(E, F)** The frequencies of CD4+ T cells and CD8+ T cells in mLN and spleen were determined by flow cytometry. **(G)** The frequency of CXCR5+ cells among CD8+ T cells in the tumor was determined by flow cytometry. **(H)** The frequency of PD-1+ cells among CD8+ T cells in the tumor was analyzed by flow cytometry. Data are presented as mean ± SD; p-values were calculated using the two-tailed Student’s t-test. *p < 0.05, **p < 0.01, ***p < 0.001, ****p < 0.0001, n.s., not significant.

We then investigated the impact of bile acid depletion on the tumor immune microenvironment. Analyzing immune cell subsets in melanomas from cholestyramine-treated mice, we observed a significant expansion of both CD4^+^ and CD8^+^ T cells within tumor-infiltrating lymphocytes (TILs), particularly CD8+ T cells (**Figure 4A**). Furthermore, quantitative PCR (qPCR) analysis of melanoma tissue revealed increased IFN-γ mRNA expression. Flow cytometry showed increased numbers of IFN-γ-positive CD4^+^ and CD8^+^ T cells within TILs after cholestyramine treatment (**Figure 4B**). Consistently, we also detected higher IFN-γ production in the spleen and draining lymph nodes (dLN) of cholestyramine-treated mice (**Figures 4C, D**). Flow cytometric analysis revealed that cholestyramine feeding altered the distribution of immune cells: decreasing CD4+, CD8+, and B220+ cells, while increasing CD11c+ cells, in the mesenteric lymph nodes (mLN) (**Figure 4E**; **Supplementary Figure S1A**). In contrast, cholestyramine increased the spleen’s CD4^+^ and CD8^+^ T cell populations without significantly altering the B220+ and CD11c+ cell populations (**Figure 4F**; **Supplementary Figure S1B**).

Similar trends of upregulation of CD4+ and CD8+ T cells in the spleen and mLN were observed in the 4T1 breast cancer model (**Supplementary Figures 1C, E**). Increased IFN-γ^+^ cells were also found in the spleen, not dLN, in the cholestyramine-treated 4T1 model (**Supplementary Figures 1D, F**).

We further examined T cell exhaustion, a dysfunction often observed in tumor-infiltrating T cells. Cholestyramine treatment increased CD8^+^ T cell infiltration while significantly reducing the PD-1^+^ and CXCR5^+^ cell populations within CD8^+^ T cells in 4T1 breast cancer models (**Figures 4G, H**; **Supplementary Figures S1G, H**).

Interestingly, we observed that innate lymphoid cell (iLC) subsets were also affected. iLC3 cells were upregulated in draining lymph nodes (dLN) after cholestyramine treatment, and both iLC2 and iLC3 cell frequencies were upregulated within tumors (**Supplementary Figures 1I, J**).

Next, we assessed the immune response in the MMTV-PyMT breast cancer mouse model fed with a cholestyramine diet. CD4^+^ T cells and CD8^+^ T cells were upregulated in the spleen, mLN, and Tumor (**Supplementary Figures 2A, C, E**). Higher IFN-γ production was shown in the spleen, mLN, and Tumor (**Supplementary Figures 2B, D, F**). These comprehensive immunological analyses demonstrate that bile acid depletion promotes a robust anti-tumor immune response characterized by increased T cell infiltration, activation, and reduced exhaustion.

### Bile acids stimulate FGFR2 and PD-L1 expression in the tumor

**Figure 5:**
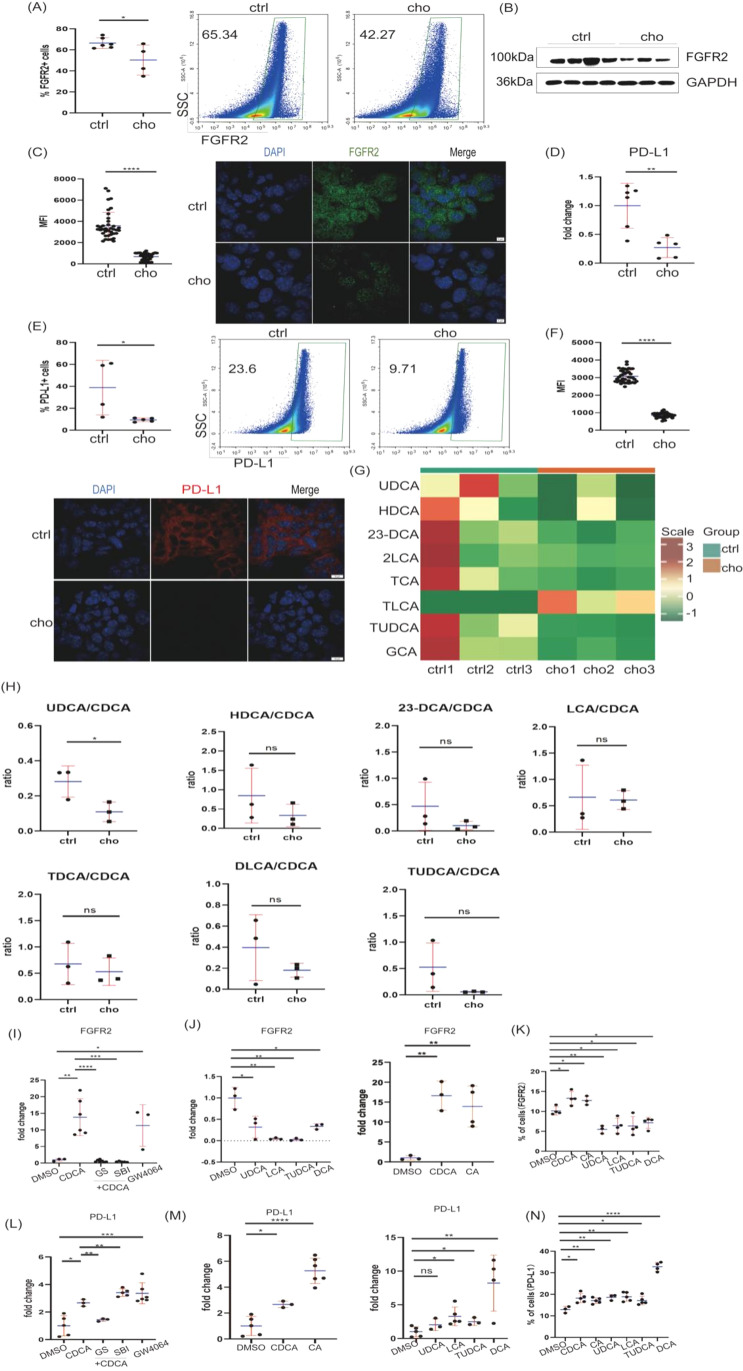
Bile acids stimulate FGFR2 and PD-L1 expression in melanoma cells. **(A)** FGFR2 expression levels in melanoma were analyzed by flow cytometry. **(B)** FGFR2 expression levels were determined by Western blot in melanomas. **(C)** Melanomas were stained with DAPI(blue) and FGFR2(green), permeabilized with 0.3% TritonX-100 in blocking reagent, and analyzed by confocal imaging. The scale bars are 5 μm. **(D)** PD-L1 mRNA level was determined by RT-qPCR in melanomas. **(E)** PD-L1 expression level was determined by flow cytometry in melanomas. **(F)** Melanomas were stained with DAPI (blue) and PD-L1(red) and analyzed by confocal imaging. The scale bars are 10μm. **(G, H)** C57BL/6J mice were fed 2% cholestyramine or a regular diet. A week later, mice were injected subcutaneously with 0.5 × 10^^6^ B16-OVA cells on the left lower back. UPLC-MS analyzed bile acid profiles in melanomas. The hierarchical clustering datasets were processed in R, with a particular focus on the ratio of secondary bile acids to primary bile acids. **(I)** CDCA, CDCA+Guggulsterone, and CDCA+SBI-115 stimulated B16 cells, and RT-qPCR determined the FGFR2 mRNA expression level. **(J)** CDCA, CA, UDCA, LCA, TUDCA, DCA, and FGFR2 stimulated B16 cells. RT-qPCR examined the expression level. **(K)** B16 cells were incubated with CDCA, CA, UDCA, LCA, TUDCA, and DCA, and FGFR2-positive cells were analyzed by flow cytometry. **(L)** CDCA, CDCA+Guggulsterone, and CDCA+SBI-115 stimulated B16 cells, and RT-qPCR determined PD-L1 mRNA expression level. **(M)** B16 cells were stimulated by CDCA, CA, UDCA, LCA, TUDCA, and DCA, and RT-qPCR was used to examine the PD-L1 mRNA expression level. **(N)** B16 cells were incubated with CDCA, CA, UDCA, LCA, TUDCA, and DCA, and PD-L1-positive cells were analyzed by flow cytometry. Data are presented as mean ± SD; p-values were calculated using the two-tailed Student’s t-test. *p < 0.05, **p < 0.01, ***p < 0.001, ****p < 0.0001, n.s., not significant.

Genome-wide microarray analysis in human melanomas identified FGFR2 as a potential biomarker (15). We, therefore, investigated the expression of FGFR2 and PD-L1, an immunosuppressive ligand, in response to bile acids. Cholestyramine treatment downregulated FGFR2 mRNA expression in melanoma mouse models (**Supplementary Figure 3**). Reduced FGFR2 expression was further confirmed at the protein level by flow cytometry, western blot, and immunofluorescence (**Figures 5A–C**). Consistent with previous reports linking FGFR2 and PD-L1 (16–18), we also observed decreased mRNA and protein levels of PD-L1 following cholestyramine treatment in melanomas (**Figures 5D–F**) and similarly in the 4T1 breast cancer model (**Supplementary Figures 4A–G**).

To identify the bile acid receptor mediating these effects, we stimulated B16 cells with chenodeoxycholic acid (CDCA), a primary bile acid known to activate FXR and TGR5. CDCA upregulated FGFR2 expression, an effect partially reversed by the FXR antagonist guggulsterone and the TGR5 antagonist SBI-115 (**Figure 5I**) and potentiated by the FXR agonist GW4064. However, while CDCA increased PD-L1 expression, this effect was specifically blocked by the FXR antagonist guggulsterone, but not by the TGR5 antagonist (**Figure 5L**). Similar results were obtained in human breast cancer MCF-7 cells, where CDCA increased FGFR2 and PD-L1 expression via FXR activation (**Supplementary Figures 4H–K**). These data indicate that bile acids stimulate FGFR2 and PD-L1 expression in tumor cells, with FGFR2 potentially regulated by FXR and TGR5, and PD-L1 primarily mediated through FXR.

### Bile acid profile in tumor tissues after cholestyramine treatment

To understand how cholestyramine impacts the bile acid milieu in the TME, we analyzed bile acid profiles in tumors from cholestyramine-treated mice. UPLC-MS analysis revealed that cholestyramine treatment effectively reduced the levels of several primary bile acids in melanomas, including TCA and GCA, as well as secondary bile acids such as UDCA, HDCA, TUDCA, 23-DCA, and iLCA (**Figure 5G**). Interestingly, TLCA levels were upregulated (**Figure 5G**). Analysis of bile acid ratios showed a decrease in the secondary/primary bile acid ratio in cholestyramine-treated tumors (**Figure 5H**). Glycine-conjugated bile acids (GHDCA, GLCA, GDHCA, GDCA) were generally increased after cholestyramine treatment, while taurine-conjugated bile acids (Tω-MCA, Tα-MCA, TUDCA) were decreased in the MMTV-PyMT mice model (**Supplementary Figure 2G**). *In vitro*, primary bile acids (CDCA, CA) upregulated FGFR2 and PD-L1 expression in B16 cells, whereas secondary bile acids downregulated FGFR2 but upregulated PD-L1 (**Figures 5J, K, M, N**; **Supplementary Figures S4L–O**). This detailed bile acid profiling demonstrates that cholestyramine treatment effectively alters the tumor bile acid landscape, particularly by reducing overall bile acid concentration and shifting the balance of primary and secondary bile acids, thereby differentially affecting FGFR2 and PD-L1 expression.

### Bile acids activate retinoic acid response via FXR-RARα interaction, sustaining aerobic glycolysis

**Figure 6:**
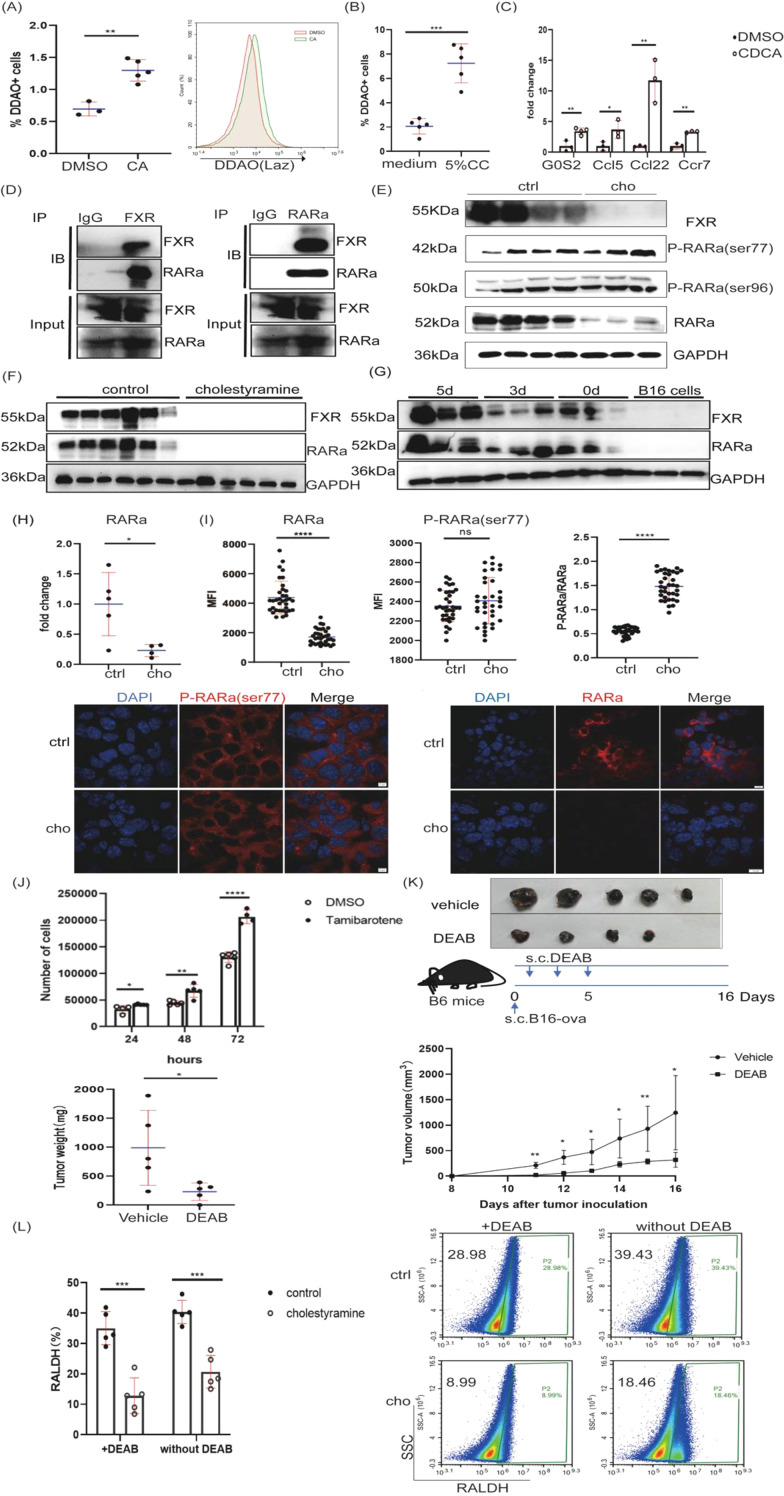
Bile acid activates retinoic acid response via FXR-RARα interaction, inhibiting RA and FXR signaling. **(A)** CA modulates RA signaling from bone marrow cells from RA reporter mouse stimulated bone marrow cells, and DDAO fluorescent signals were analyzed by flow cytometry. **(B)** Bone marrow cells from RA reporter mice were stimulated with 5% cecum content. DDAO fluorescent signals were analyzed by flow cytometry. **(C)** CDCA stimulated B16 cells, and RT-qPCR analyzed retinoic acid signaling-related gene expression levels. **(D)** Melanoma cells from C57BL/6J mice were injected with B16 cells and then lysed; FXR and RARα antibodies were used for co-IP. **(E)** FXR, RARα, and P-RARα(ser77) or P-RARα(ser96) expression was analyzed by western blot in melanomas. **(F)** FXR and RARα expression levels were determined by western blot in the MMTV-PyMT mouse breast cancer model. **(G)** C57BL/6J mice were subcutaneously injected with 0.5 × 10^6 B16 cells on the left lower back on 0 days, the third day, and the fifth day. Melanoma cells and B16 cells were lysed, and FXR and RARα antibodies were used for western blot analysis. **(H)** RT-qPCR was used to assess RARα mRNA expression in melanomas. **(I)** Melanomas were stained with DAPI (blue), RARα(red), and either P-RARA (ser77) (red) antibody and analyzed by confocal imaging. The scale bars in this figure are 10 μm and 5 μm. **(J)** B16 cells were stimulated with Tamibarotene 10μM for 24 hours, 48 hours, or 72 hours. Cell numbers were analyzed by flow cytometry. **(K)** Wild-type C57BL/6J mice (5–6 weeks, male) were divided into two groups(n=5). The mice were injected subcutaneously with 0.5 × 10^^6^ B16-OVA cells on the left lower back. Each mouse was injected subcutaneously with a dose of DEAB (2mg) every other day, and 11 days later, the mice were sacrificed. Representative melanoma images are shown. **(L)** RALDH expression levels were analyzed by flow cytometry in melanomas. Data are presented as mean ± SD; p-values were calculated using the two-tailed Student’s t-test. *p < 0.05, **p < 0.01, ***p < 0.001, ****p < 0.0001, (n) s., not significant.

We explored potential downstream signaling mechanisms, focusing on retinoic acid (RA) signaling, as BAs are known to activate the retinoic acid response in other contexts (19). We used RA-reporter transgenic mice to stimulate bone marrow cells with cholic acid (CA) *in vitro*. CA activated the RARE reporter (DDAO, LacZ) in bone marrow cells (**Figure 6A**). Cecum content, enriched in bacteria-metabolized bile acids, also activated the RARE response (**Figure 6B**). CDCA stimulation of B16 cells *in vitro* also induced retinoic acid signaling-related genes (**Figure 6C**).

Given the known interaction between FXR and RARα in bile acid-mediated RA signaling in DCs, we examined whether these proteins interact in tumors. Co-immunoprecipitation (co-IP) experiments confirmed a direct interaction between FXR and RARα in melanoma tumor lysates (**Figure 6D**). Similarly, when RARα was immunoprecipitated, the interaction between FXR and RARα was confirmed with an FXR antibody (**Figure 6D**).

We observed reduced protein levels of FXR and RARα in cholestyramine-treated melanoma tumors and MMTV-PyMT breast cancer models (**Figures 6E, F**). Furthermore, tumor-intrinsic FXR and RARα expression was higher *in vivo* than in B16 cells cultured *in vitro* (**Figure 6G**). Cholestyramine treatment downregulated total RARα levels, but not phosphorylated RARα (**Figures 6E, H, I**). Cholestyramine also reduced RARE response in reporter cells derived from mLN, though it did not alter immune cell frequency in mLN (**Supplementary Figures 5A, B**).

Furthermore, to clarify whether retinoic acids are involved in tumor growth. We stimulated B16 cells with Tamibarotene *in vitro*, a synthetic retinoid more stable than all-trans retinoic acid. Tamibarotene enhanced B16 cell proliferation (**Figure 6J**), and DEAB, an inhibitor of RA synthesis, reduced melanoma tumor growth *in vivo* (**Figure 6K**). RALDH expression, regulated by RA signaling and associated with cancer progression (20), was decreased by cholestyramine treatment (**Figure 6L**).

**Figure 7:**
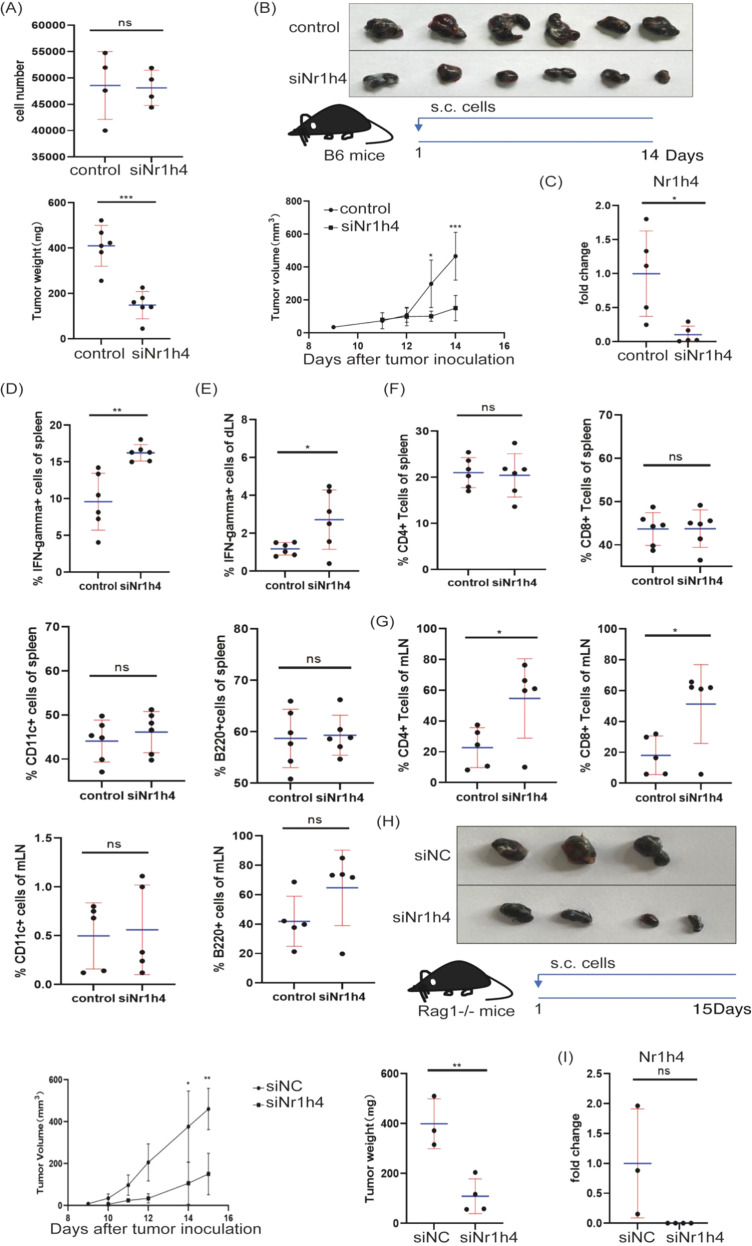
Nr1h4 is essential for tumor growth. **(A)** B16 cells were transfected with siNr1h4 for 28 hours. Cell numbers were analyzed by flow cytometry. **(B)** Wild-type C57BL/6J mice (8-9weeks, male) were divided into two groups(n=6). The mice were given a subcutaneous injection of 0.5 × 10^^6^ B16 cells that had been transfected for 48 hours into the left lower back. Thirteen days later, the mice were sacrificed. Representative melanoma images are shown. **(C)** Nr1h4 mRNA expression level was determined by RT-qPCR in the melanoma model. **(D, E)** IFN-γ^+^ cell frequency in the spleen and dLN was analyzed by flow cytometry. **(F, G)** The frequencies of CD4+ T cells, CD8+ T cells, CD11c+ cells, and B220+ cells in the spleen and mesenteric lymph nodes (mLN) were determined by flow cytometry. **(H)** Rag1^-/-^mice (3–4 weeks, male) were divided into two groups (siNC=3, siNr1h4 = 4). The mice were given a subcutaneous injection of 0.5 × 10^^6^ B16 cells transfected for 48 hours into the left lower back. About half a month later, the mice were sacrificed. Representative melanoma images are shown. **(I)** Nr1h4 mRNA expression level was determined by RT-qPCR in the Rag1^-/-^ mouse melanoma model. Data are presented as mean ± SD; p-values were calculated using the two-tailed Student’s t-test. *p < 0.05, **p < 0.01, ***p < 0.001, ****p < 0.0001, n.s., not significant.

**Figure 8:**
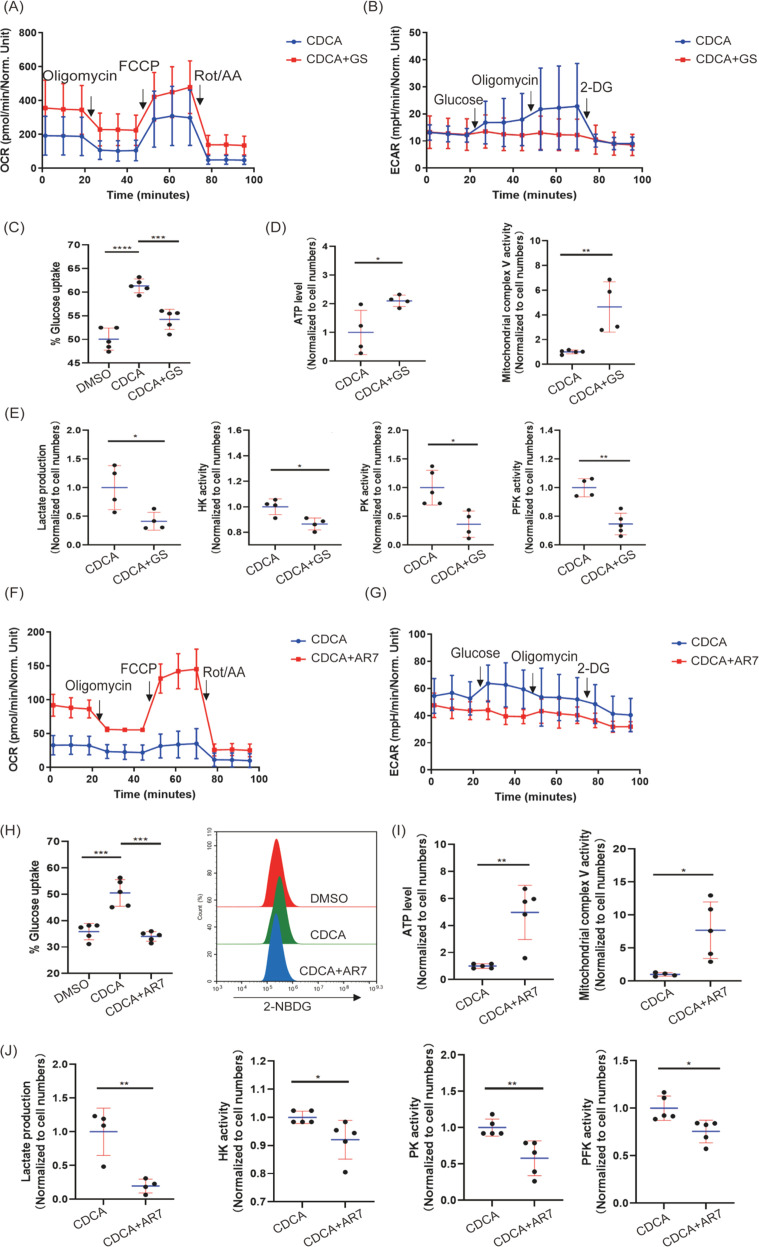
Bile acid induces tumor aerobic glycolysis via FXR-RARα interactions. **(A, B)** B16 cells were stimulated with CDCA (10μM) and Guggulsterone(10μM), and OCR and ECAR were measured using Seahorse XFe24 Analyzer. **(C)** B16 cells were stimulated with CDCA (10μM) and Guggulsterone (10μM). Subsequently, the cells were stained with 2-NBDG in a No-Glucose medium, and glucose uptake was examined by flow cytometry. **(D, E)** B16 cells were stimulated with CDCA (10μM) and Guggulsterone(10μM). ATP level, mitochondrial complex V activity, lactate production, HK activity, PK activity, and PFK activity were measured by a microplate reader. **(F, G)** B16 cells were stimulated with CDCA (10μM) and AR7(1μM), and OCR and ECAR were measured using Seahorse XFe24 Analyzer. **(H)** B16 cells were stimulated with CDCA (10μM) and AR7 (1μM). Subsequently, the cells were stained with 2-NBDG in a No-Glucose medium and examined for glucose uptake by flow cytometry. **(I, J)** B16 cells were stimulated with CDCA (10μM) and AR7 (1μM). Subsequently, ATP levels, mitochondrial complex V activity, lactate production, HK activity, PK activity, and PFK activity were measured using a microplate reader. Data are presented as mean ± SD; p-values were calculated using the two-tailed Student’s t-test. *p < 0.05, **p < 0.01, ***p < 0.001, ****p < 0.0001, n.s., not significant. .

Moreover, knockdown of FXR using siRNA *in vitro* did not affect B16 cell proliferation (**Figure 7A**) but diminished growth *in vivo* (**Figure 7B**), with reduced FXR protein confirmed (**Figure 7C**). FXR knockdown also increased IFN-γ positive immune cells (**Figures 7D, E**) and CD4+ and CD8+ T cell infiltration in mLN (**Figure 7G**) without altering T cell frequency in spleen (**Figures 7F, G**). Transplantation of FXR-knockdown B16 cells into Rag1^-/-^ mice (lacking T and B cells) still showed reduced tumor growth (**Figures 7H, I**), suggesting a tumor cell-intrinsic role for FXR. Finally, bile acid (CDCA) induced and stabilized the FXR-RARα complex (**Figures 6D–G**). Subsequent metabolic analysis revealed that glucose uptake, lactate production, and activities of HK, PK, and PFK were decreased upon FXR or RARα inhibition using guggulsterone or AR7, respectively (**Figures 8B, C, E, F–J**), while ATP levels and OCR increased (**Figures 8A, D**). These data converge to demonstrate that bile acids activate retinoic acid signaling by interacting with FXR and RARα, promoting tumor aerobic glycolysis and contributing to tumor progression.

## Discussion

This study provides compelling evidence for a novel role of microbiota-metabolized bile acids (BAs) as critical drivers of tumor progression by orchestrating a metabolic and immunological axis within the tumor microenvironment (TME). Our findings demonstrate that BAs are enriched in solid tumors and directly promote tumor aerobic glycolysis, a hallmark of cancer metabolism, while suppressing anti-tumor immunity. Mechanistically, we have elucidated that this dual effect is mediated by bile acid activation of the FXR-RARα receptor complex, a signaling pathway that emerges as a key regulator of tumor metabolism and immune evasion in the TME.

Cancer is a complex and multifaceted disease (21), and metabolic reprogramming is now recognized as a core hallmark contributing to its initiation, progression, and therapeutic resistance. While genomic mutations play a pivotal role in tumor development, recent deep-sequencing studies have revealed that somatic mutations are surprisingly abundant even in normal tissues, suggesting that mutations alone are insufficient for malignant transformation (22–28). Non-mutagenic promoting factors, particularly those originating from the tumor microenvironment and influenced by lifestyle and environmental factors, are increasingly critical in cancer etiology and progression (29). Without any promotional factors, initiated cells could remain dormant for most of a mouse’s lifespan (30). Our findings highlight gut microbiota-derived bile acids as a critical environmental factor within the TME, driving aberrant metabolic phenotypes in tumors. We demonstrate that bile acids are enriched in the TME and fuel the energetically wasteful but biosynthetically advantageous process of aerobic glycolysis in tumor cells. This is consistent with the established role of altered metabolic phenotypes relative to their normal tissue counterparts, in which fast-growing tumors characteristically exploit aerobic glycolysis to support anabolic growth and evade immune surveillance (1–3). Our work underscores that bile acids actively sustain this aberrant glycolytic phenotype within the TME.

Beyond their metabolic effects, our study reveals a crucial link between bile acids and tumor-associated immunosuppression. Depletion of bile acids dramatically alters the TME immune landscape, promoting increased infiltration of cytotoxic T cells, enhancing IFN-γ production, and reducing T cell exhaustion. These immunological changes directly correlate with the observed suppression of tumor growth. This is particularly relevant, as accumulating evidence underscores that tumor aerobic glycolysis establishes an immunosuppressive environment within the TME, impeding the efficacy of checkpoint blockade and adoptive T-cell therapy (5, 6, 31). Our data extend this concept by identifying bile acids as key TME components that contribute to this immunosuppressive condition, suggesting that targeting bile acid-driven metabolic reprogramming may synergize with and improve immunotherapeutic outcomes. The intricate interplay between metabolism and immunity in the TME is increasingly recognized as a critical determinant of anti-tumor responses (32).

A key novelty of our study lies in elucidating the FXR-RARα receptor interaction as the central mechanistic axis mediating the pro-tumorigenic effects of bile acids. We demonstrate that bile acids activate retinoic acid signaling in tumor cells through this synergistic receptor complex. FXR, a well-established bile acid sensor, and RARα, a retinoic acid receptor crucial in various cellular processes, converge to form a functional unit that appears to drive metabolic and immune reprogramming within the TME. The cooperation between FXR and RARα in this context is particularly noteworthy. While both receptors are ligand-activated transcription factors, their interaction and synergistic action in mediating bile acid-induced effects in tumor cells and the TME represent a novel finding. This receptor interplay likely enables fine-tuned and coordinated regulation of downstream target genes involved in both glycolysis and immune modulation, underscoring the complexity of bile acid signaling in cancer. Our findings further suggest that this FXR-RARα axis is essential for sustaining bile acid-driven aerobic glycolysis, as inhibition of either receptor component effectively reverses the glycolytic shift and promotes a more oxidative metabolic state in tumor cells.

Identifying bile acids and the FXR-RARα axis as drivers of tumor aerobic glycolysis and immune suppression holds significant translational potential. Given that targeting aerobic glycolysis and reversing tumor immunosuppression are considered promising therapeutic strategies for cancer (33), our findings suggest a novel avenue for intervention. As we demonstrated using cholestyramine, depletion of bile acids could represent a strategy to reduce tumor glycolysis, enhance anti-tumor immunity, and thereby inhibit tumor growth. Furthermore, targeting the FXR-RARα interaction directly, perhaps through pharmacological inhibitors or modulators of this receptor complex, could offer a more specific and targeted therapeutic approach. Considering that bile acids are increasingly implicated in various gastrointestinal and hepatobiliary cancers (9, 10) and other cancer types, as shown in our study, targeting this pathway may have broad applicability across different malignancies. Moreover, the identified link between bile acids, FGFR2, and PD-L1 expression provides further rationale for investigating FGFR2-targeted therapies, particularly in tumors with high bile acid content. The observation that bile acids modulate the expression of these clinically relevant targets highlights the potential to modulate the bile acid pathway to impact tumor biology and treatment responses.

While this study provides compelling evidence, some limitations and future research directions should be considered. Firstly, while we focused on melanoma and breast cancer models, further studies are needed to validate these findings in a broader range of cancer types and human tumor samples. Secondly, although we elucidated the involvement of FXR-RARα, the precise downstream target genes and signaling pathways mediating this receptor complex’s effects on glycolysis and immune regulation in the TME require further in-depth investigation. Future research should also explore the specific microbiota populations responsible for producing tumor-promoting bile acid species and investigate whether dietary or microbiome-modulating strategies can reduce intratumoral bile acid levels. Finally, preclinical studies evaluating the efficacy of bile acid-modulating agents, FXR-RARα inhibitors, or combination therapies targeting this pathway in conjunction with immunotherapy in more clinically relevant models are warranted to translate these findings toward improved cancer therapies.

In conclusion, our study reveals a critical, previously underappreciated role for microbiota-derived bile acids in promoting tumor aerobic glycolysis and orchestrating immune evasion via the FXR-RARα signaling axis. These findings identify the bile acid-FXR-RARα pathway as a potential therapeutic vulnerability in cancer, offering new avenues for targeted metabolic and immunomodulatory interventions. By highlighting the interplay between bile acid metabolism, tumor metabolism, and anti-tumor immunity, this research advances our understanding of the complex tumor microenvironment and opens promising perspectives for improving cancer diagnosis and treatment.

## Methods

### Animal experiments

The Tg(RARE-Hspa1b/lacZ)12Jrt/J strain was obtained from Jackson Laboratories and maintained as homozygotes to retain the reporter activity. Male C57BL/6J (5–9 weeks old) mice and female BALB/c (4–5 weeks old) mice were purchased from Chongqing Tengxin Biotechnology. Male Rag1^-/-^ (3–4 weeks old) mice and female MMTV-PyMT (5–6 weeks old) mice were purchased from GemPharmatech. The Institutional Animal Care and Use Committee at Southwest Medical University approved all research animal protocols.

### Diets source

The regular diet (#1010062) was purchased from Jiangsu Xietong Pharmaceutical Bio-engineering Co., Ltd. Cholestyramine resin was purchased from Sigma (#C302192), and a 2% cholestyramine diet was prepared at Jiangsu Xietong Pharmaceutical Bio-engineering Co., Ltd.

### Mouse models

The C57BL/6J mice were subcutaneously injected with 0.5 × 10^6 B16 cells on the left lower back on days 0, 3, and 5. The tumor in each mouse was harvested using UPLC-MS and Western blot analysis. The C57BL/6J mice were fed either a 2% cholestyramine diet or a regular diet for a week, and melanoma models were established by subcutaneous injection of 0.5 × 10^6 B16-OVA cells or B16 cells in 100 μL of PBS into the left lower back. About half a month after injection, each mouse’s tumor was harvested for measurement, UPLC-MS, flow cytometry, western blot, RT-qPCR, confocal imaging, and RNA sequencing. The BALB/c mice were fed with 2% cholestyramine or a regular diet for a week, and 4T1 breast cancer models were established by subcutaneous injection of 2.5 × 10^^5^ tumor cells in 100 μL of PBS in the left lower back. About half a month after injection, each mouse’s tumor was harvested for measurement, flow cytometry, western blot, RT-qPCR, and confocal imaging. The MMTV-PyMT mice were fed either 2% cholestyramine or a regular diet for a week; approximately nineteen days later, each mouse’s tumor was harvested for measurement, flow cytometry, Western blot, and UPLC-MS analysis. C57BL/6J mice were injected subcutaneously with 0.5 × 10^^6^ B16-OVA cells on the left lower back. Each mouse was injected with a dose of DEAB (2mg) three times every other day via subcutaneous injection. Eleven days later, each mouse’s tumor was harvested for measurement. The Rag1^-/-^mice were given a subcutaneous injection of 0.5 × 10^^6^ B16 cells transfected within 48 hours on the left lower back. About half a month later, each mouse’s tumor was harvested for RT-qPCR measurement. Tumor volume (TV) was calculated using the formula: TV (mm^3^)=d^2^ × D/2, where d and D are the shortest and the longest diameter, respectively. TV<2000mm^3^, d<20mm, D<20mm.

### Cell lines

The mouse melanoma cell lines B16 and B16-OVA were obtained from the American Type Culture Collection (ATCC). The mouse mammary cancer cell line 4T1 was obtained from Shanghai Zhongqiao Xin Zhou Biotech, and the human breast cell line MCF-7 was obtained from the Laboratory of Oncology at the Affiliated Hospital of Southwest Medical University. The B16 cells, B16-OVA cells, and 4T1 cells were cultured in RPMI 1640 medium (Hyclone) supplemented with 1% penicillin-streptomycin (Thermo Fisher Scientific) and 10% FBS. The MCF-7 cells were cultured in DMEM (Hyclone) supplemented with 1% antibiotics and 10% FBS.

### Reagents

The following reagents were purchased: DDAOG (F1179, Invitrogen); lithocholic acid (L106779, Aladdin-e), cholic acid (C103692, Aladdin-e), chenodeoxycholic acid (C104902, Aladdin-e), ursodeoxycholic acid (U110695, Aladdin-e), deoxycholic acid(D103697, Aladdin-e), tauroursodeoxycholic acid(T303865, Aladdin-e), and tamibarotene (T427039,Aladdin-e), AR7 (SML0921,Sigma), DEAB (D86256,Sigma), Guggulsterone(HY-107738,MCE), SBI-115(HY-111534,MCE),GW4064 (HY-50108,MCE), 2-NBDG (M6327,AbMole).

### FACS and antibody

Cancer cells were washed and suspended in FACS buffer (2% FBS in PBS), incubated with fluorochrome-coupled agents for 30 min at 4 °C, and then washed in FACS buffer. Data were obtained with the Acea NovoCyte series flow cytometer and analyzed using FlowJo software v9.6 (FlowJo, Ashland, OR, USA). The following anti-murine antibodies were used for flow cytometry: CD4 (RM4-5, Biolegend), CD8(53-6.7, Biolegend), IFN-γ(XMG1.2, Biolegend), CD45(30-F11, Biolegend), PD-1(RMP1-30, Biolegend), T-bet(4B10, Biolegend), GATA3(6E10A23, Biolegend), CD11c(N418,Invitrogen),RORγt(B2D,Invitrogen),B220(RA3-6B2,ebioscience),CXCR5(SPRCL5,ebioscience),PD-L1(CD274,B7-H1)(MIH5,ebioscience).Anti-rabbit antibody:FGFR2 (A12436, ABclonal).

### Bone marrow cell isolation

Bone marrow cells were obtained from 5–8 weeks-old RA reporter mice. Briefly, bone marrow cells were flushed out from the femurs and tibias. Bone marrow cells were cultured in a complete culture medium (RPMI 1640 supplemented with 10% FBS, 25 mM HEPES, 5 mM β-ME, and 1% antibiotics).

### Tumor-infiltrating lymphocyte isolation

Melanomas from mice were isolated and finely cut. Tumor samples were digested twice for 25 minutes each at 37 °C on a shaking platform with a digestion buffer containing RPMI 1640 Medium, Collagenase/Hyaluronidase (#07912, StemCell), and DNase I Solution (#100-0762, StemCell). Use a 70um nylon mesh strainer with the recommended medium, centrifuge, and discard the supernatant. Add Ammonium Chloride Solution (#07800, StemCell) and incubate at room temperature for 5 minutes. Then, top up with the recommended medium, centrifuge, and discard the supernatant. TIL cells were purified using magnetic-activated cell sorting with the EasySep™ mouse CD45 positive selection kit (#100-0350, StemCell). Isolated cells were stained with antibodies against CD4, CD8, and IFN-γ on ice for 30min, followed by FACS staining.

### RALDH assay

Mice’s cancer cells were mechanically digested. According to the manufacturer’s instructions, they used the ALDEFLUOR™ Kit (#01700, StemCell).

### β-galactosidase activity

Bone marrow cells from RA reporter mice were incubated with the galactosidase substrate DDAO galactoside (DDAOG)(10μM) in serum-free Hank’s buffer for 0.5-2h at 37 °C. The cells were washed three times with Hank’s buffer before flow cytometry to measure the signal from DDAOG-to-DDAO conversion, which can be excited with a 633 nm laser (excitation/emission maxima ~645/660 nm).

### Quantitative real-time PCR

Tumor tissues were ground into single cells. Total RNA was extracted using the Total RNA Isolation Kit (RC101-01, Vazyme). cDNA was synthesized with a cDNA Synthesis Kit (R323-01, Vazyme). Real-time PCR was performed using the LightCycler480 (Roche) and SYBR Green Master Mix (Q711-02, Vazyme). Gene expression was normalized to GAPDH and calculated using the ΔΔCt method. PCR primers of mouse and human genes were purchased from OriGene. GAPDH was used as an internal control.

### Co-IP and Western blot

All experimental producers were instructed by the manufacturer’s instructions of the immunoprecipitation Kit (10007D, Invitrogen). Tumor tissues were collected and lysed on ice with RIPA Buffer (#9806S, CST) containing Protease/Phosphatase Inhibitor Cocktail (#5872S, CST) and 1 mM PMSF (ST505, Beyotime). Cell extracts were loaded onto Biofuraw™ Precast Gel (D0174S, Beyotime), separated by electrophoresis, and transferred onto NC membranes (Millipore). Signals were generated with High-sig ECL Western Blotting Substrate (4AW011-200, 4A Biotech). The antibodies: RARα mouse mAb (sc-515796, Invitrogen) or RARα rabbit mAb (E6Z6K, CST), Phospho-RARA (Ser77) rabbit mAb (P45-99142, Invitrogen), Phospho-RARA (Ser96) rabbit mAb (P45-64622, Invitrogen), GAPDH mouse mAb (AC002, ABclonal), FXR/NR1H4 mouse mAb (E4B8P, CST), and FGFR2 rabbit mAb (A12436, ABclonal). Band signals were quantified in ImageJ and analyzed in GraphPad Prism.

### Immunofluorescence staining

Tumors were carefully isolated, embedded in optimal cutting temperature compound, and snap-frozen in liquid nitrogen. Cryosections, 5 μm thick, were fixed for 30 min at 4 °C with 4% PFA. Then incubated with the following primary antibodies: rabbit anti-FGFR2 (A12436, ABclonal), mouse PE anti-CD274 (MIH5, ebioscience), COL1A2 rabbit pAb (A5786, ABclonal), COL3A1(A3795, ABclonal); secondary antibody: goat anti-rabbit IgG (A11034, Invitrogen). DAPI (4′, 6-diamidine-2-phenylindole) staining of the nucleus. Images on an Olympus FV1000 microscope equipped with constant exposure. Maximum intensity projections are presented.

### UPLC-MS analysis of BAs

Tumor tissue samples were collected. BAs were detected on UPLC (Shim-pack UFLC SHIMADZU CBM30A) and MS/MS (Applied Biosystems 4500 QTRAP). The analytical conditions were as follows: HPLC column, waters ACQUITY UPLC HSS T3 C18 (1.8μm, 2.1 mm*100 mm); solvent system, water (0.01% acetic acid and 5mmol/L ammonium acetate): acetonitrile (0.01% acetic acid); gradient program, 95:5 V/V at 0 min, 60:40 V/V at 0.5 min,50:50 V/V at 4.5 min, 25:75 V/V at 7.5 min, 5:95 V/V at 10 min, 95:5 V/V at 12 min; flow rate, 0.35 ml/min; temperature, 40 °C; and injection volume: 3μl. The effluent was alternately connected to an ESI-triple quadrupole-linear ion trap (QTRAP)-MS. The UPLC-MS datasets for hierarchical 415-cluster analysis were processed in R (https://www.r-project.org/).

### RNA sequencing and analysis

Tumor tissues were collected from mice, and the products were subjected to RNA isolation, library construction, and sequencing analysis by MetWare Biotechnology Co., Ltd. (Wuhan, China).

### Cellular respiration and extracellular acidification assays

OCR and ECAR measurements were performed using the Seahorse XF24 Extracellular Flux Analyzer (Seahorse, USA). After trypsinization, cancer cells were seeded in Seahorse XF24 cell plates (1.2 × 10^^4^ cells/well) in a humidified incubator at 37 °C and 5% CO2 for 24–48 h. The cell plates were then placed in a 37 °C, 0% CO2 incubator for 60 minutes prior to the start of the assay. According to the manufacturer’s protocols, a glycolytic stress test kit (103020, Seahorse) and a mitochondrial stress test kit (103015, Seahorse) were used to measure glycolytic and mitochondrial metabolic flux.

### Glucose uptake assay

Cells were seeded in six-well plates (2 × 10^^5^ cells/well) and incubated at 37 °C for 24 h. The cell medium was then changed to a glucose-free medium with or without fluorescent 2-NBDG. The cells were incubated at 37 °C for an additional 1 hour, washed with PBS, and digested into single cells for analysis.

### Lactate production, ATP level, HK activity, PK activity, PFK activity, and mitochondrial complex V activity measurements

The production of lactate and the level of ATP by cancer cells were measured using a lactate assay kit (BC2235, Solarbio) and an ATP assay kit (BC0305, Solarbio). The activities of the HK activity assay kit (BC0745, Solarbio), PK activity assay kit (BC0545, Solarbio), PFK activity assay kit (BC0535, Solarbio), and mitochondrial complex V activity assay kit (BC1445, Solarbio) were performed according to the manufacturer’s protocols.

### siRNA assay

The siRNA (Nr1h4) kit was purchased from RiboBio Co., Ltd. (Guangzhou, China) (SIGS0115363-4) and transfected into B16 cells according to the manufacturer’s instructions. The mice were given a subcutaneous injection of 0.5 × 10^^6^ cells in 100 μL of PBS on the left lower back. On day 13, after injection, the tumor in each mouse was harvested for measurement.

### Sirius Red staining

All experimental producers were instructed according to the manufacturer’s instructions for the Sirius Red staining kit. The kit was purchased from SenBeiJia Biological Technology Co., Ltd. (Nanjing, China) (BP-DL030). The collagen content was measured using ImageJ.

### Statistical analysis

The statistical analysis was performed with GraphPad Prism software. All plot graphs show means with standard deviation (SD). Statistical significance was determined with the two-tailed Student’s t-test. A p-value of less than 0.05 was considered statistically significant. *p< 0.05, **p<0.01, ***p< 0.001, not significant (N.S.). Data that did not exhibit a normal distribution were analyzed using the nonparametric Kruskal-Wallis test, followed by Dunn’s *post hoc* test.

## Data Availability

The data presented in the study are deposited in the NCBI Sequence Read Archive (SRA) repository, accession number PRJNA631431.

## Supplementary material

Supplementary Figure 1 Depleting bile acid stimulates an anti-tumor immune response. **(A, B)** The frequencies of CD11c+ and B220+ cells in the mesenteric lymph nodes (mLN) and spleen of the melanoma mouse model were analyzed by flow cytometry. **(C, D)** The frequencies of CD4+ T cells, CD8+ T cells, CD11c+ cells, B220+ cells, and IFN-γ+ cells in the spleens of 4T1 breast cancer mice were analyzed by flow cytometry. **(E)** CD4^+^ T cells, CD8^+^ T cells, CD11c^+^ cells, and B220^+^ cells frequency in mLN were determined by flow cytometry in the 4T1 breast cancer mouse model. **(F)** IFN-γ^+^ cell frequency in dLN was analyzed by flow cytometry in the 4T1 breast cancer mouse model. **(G, H)** The frequency of CD8+ T cells in the melanoma mouse model was determined by flow cytometry. **(I, J)** CD4^+^ T cells, RORγt+ cells, T-bet+ cells, and GATA3+ cells were quantified in the draining lymph node (dLN) and tumor by flow cytometry. Data are presented as mean ± SD; p-values were calculated using the two-tailed Student’s t-test. *p < 0.05, **p < 0.01, ***p < 0.001, ****p < 0.0001, n.s., not significant.

Supplementary Figure 2 The effect of depleting bile acid on immune cells and bile acid in the MMTV-PyMT mouse breast cancer model. **(A)** CD4^+^ T cells, CD8^+^ T cells, CD11c^+^ cells, and B220^+^ cells frequency in the spleen were analyzed by flow cytometry. **(B)** IFN-γ^+^ cell frequency in the spleen was analyzed by flow cytometry. **(C)** The frequency of CD4+ T cells, CD8+ T cells, CD11c+ cells, and B220+ cells in mLN was determined by flow cytometry. **(D)** IFN-γ^+^ cells frequency in mLN was analyzed by flow cytometry. **(E)** The frequency of CD4+ T cells, CD8+ T cells, CD11c+ cells, and B220+ cells in the tumor was examined by flow cytometry. **(F)** IFN-γ^+^ cells frequency in the tumor was analyzed by flow cytometry. **(G)** MMTV-PyMT mice were fed either a 2% cholestyramine diet or a regular diet. For about 20 days, bile acid profiles in the tumor were analyzed by UPLC-MS. The hierarchical clustering datasets were processed in R. Data are presented as mean ± SD; p-values were calculated using the two-tailed Student’s t-test. *p < 0.05, **p < 0.01, ***p < 0.001, ****p < 0.0001, n.s., not significant.

Supplementary Figure 3 Melanoma biomarker expression by RT-qPCR in a melanoma model. RT-qPCR was used to examine the expression levels of biomarker genes in the melanoma model. Data are presented as mean ± SD; p-values were calculated using the two-tailed Student’s t-test. *p < 0.05, **p < 0.01, ***p < 0.001, ****p < 0.0001, n.s., not significant.

Supplementary Figure 4 Bile acids stimulate FGFR2 and PD-L1 expression in breast cancer cells. **(A)** FGFR2 expression level was analyzed by flow cytometry in 4T1 breast cancer. **(B)** RT-qPCR analyzed the FGFR2 mRNA expression level in 4T1 breast cancer. **(C)** FGFR2 expression level was determined by Western blot in 4T1 breast cancer. **(D)** Immunofluorescent analysis of FGFR2 in 4T1 breast cancer by 2% cholestyramine-fed mice. Breast cancer cells were stained with DAPI (blue) and FGFR2 (green) antibodies and analyzed by confocal imaging. The scale bars are 5μm. **(E)** PD-L1 expression level was determined by flow cytometry in 4T1 breast cancer. **(F)** PD-L1 mRNA expression level was determined by RT-qPCR in 4T1 breast cancer. **(G)** Immunofluorescent analysis of PD-L1 in 4T1 breast cancer by 2% cholestyramine-fed chow mice. Breast cancer cells were stained with DAPI (blue) and PD-L1 (red) antibodies and analyzed by confocal imaging. The scale bars are 5μm. **(H)** CDCA, CDCA+Guggulsterone, CDCA+SBI-115, and GW4064 stimulated MCF-7 cells, and RT-qPCR was used to examine FGFR2 mRNA expression levels. **(I)** CDCA, CDCA+Guggulsterone, CDCA+SBI-115, and GW4064 stimulated MCF-7 cells, and the FGFR2 expression level was examined by flow cytometry. **(J)** CDCA, CDCA+Guggulsterone, CDCA+SBI-115, and GW4064 stimulated MCF-7 cells, and RT-qPCR was used to examine PD-L1 mRNA expression levels. **(K)** CDCA, CDCA+Guggulsterone, CDCA+SBI-115, and GW4064 stimulated MCF-7 cells, and the PD-L1 expression level was examined by flow cytometry. **(L)** MCF-7 cells were stimulated by CDCA, CA, UDCA, LCA, TUDCA, and DCA, and RT-qPCR was used to analyze the FGFR2 mRNA expression level. **(M)** MCF-7 cells were incubated with CDCA, CA, UDCA, LCA, TUDCA, and DCA, and FGFR2-positive cells were analyzed by flow cytometry. **(N)** CDCA, CA, UDCA, LCA, TUDCA, and DCA stimulated MCF-7 cells, and RT-qPCR was used to analyze PD-L1 mRNA expression levels. **(O)** MCF-7 cells were incubated with CDCA, CA, UDCA, LCA, and TUDCA, and PD-L1-positive cells were analyzed by flow cytometry. Data are presented as mean ± SD; p-values were calculated using the two-tailed Student’s t-test. *p < 0.05, **p < 0.01, ***p < 0.001, ****p < 0.0001, ns., not significant.

Supplementary Figure 5 The effect of cholestyramine treatment on immune cells. **(A)** RA reporter mice were treated with cholestyramine for 4 weeks. DDAO fluorescent signals of cells (Total, CD11c^+^, CD4^+^, CD8^+^, B220^+^) from the spleen and mLN were analyzed by flow cytometry. **(B)** Total cell number from spleen and mLN, CD11c^+^ cells, CD4^+^ T cells, CD8^+^ T cells, and B220^+^ cells frequency in spleen and mLN were determined by flow cytometry. Data are presented as mean ± SD; p-values were calculated using the two-tailed Student’s t-test. *p < 0.05, **p < 0.01, ***p < 0.001, ****p < 0.0001, n.s., not significant.

Supplementary Figure 6 Expression of bile acid transporter genes during tumor growth **(A)** RT-qPCR determined OATP4, IBABP, OSTb, SLC10A2, NTCP, and OATP1 mRNA in melanomas. **(B)** SLC10A2 mRNA expression after B16 cells were transfected with siSLC10A2 for 36 hours by qPCR. **(C)** B16 cells were transfected with siSLC10A2 for 36 hours to obtain cell numbers by FCM. Data are presented as mean ± SD; p-values were calculated using the two-tailed Student’s t-test. *p < 0.05, **p < 0.01, ***p < 0.001, ****p < 0.0001, n.s., not significant.
