# Supplementary figures and images for: Bile acids enrichment fuel tumor aerobic glycolysis and immune evasion via stabilizing FXR-RARα
Source: Front Immunol. 2026 Mar 25;17:1750358. doi: 10.3389/fimmu.2026.1750358 (PMC13057493; doi:10.3389/fimmu.2026.1750358)

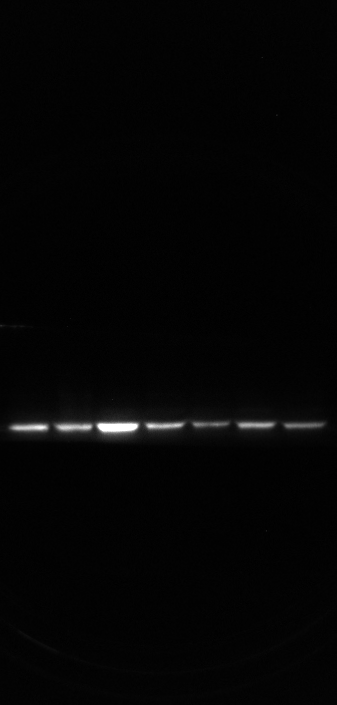

Supplement: Supplementary file 1 [file DataSheet1.zip › Fig5B fgfr2.tif]

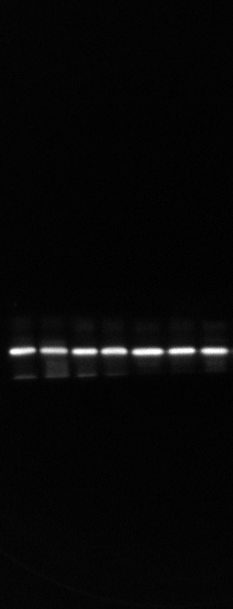

Supplement: Supplementary file 1 [file DataSheet1.zip › Fig5B gapdh-2.tif]

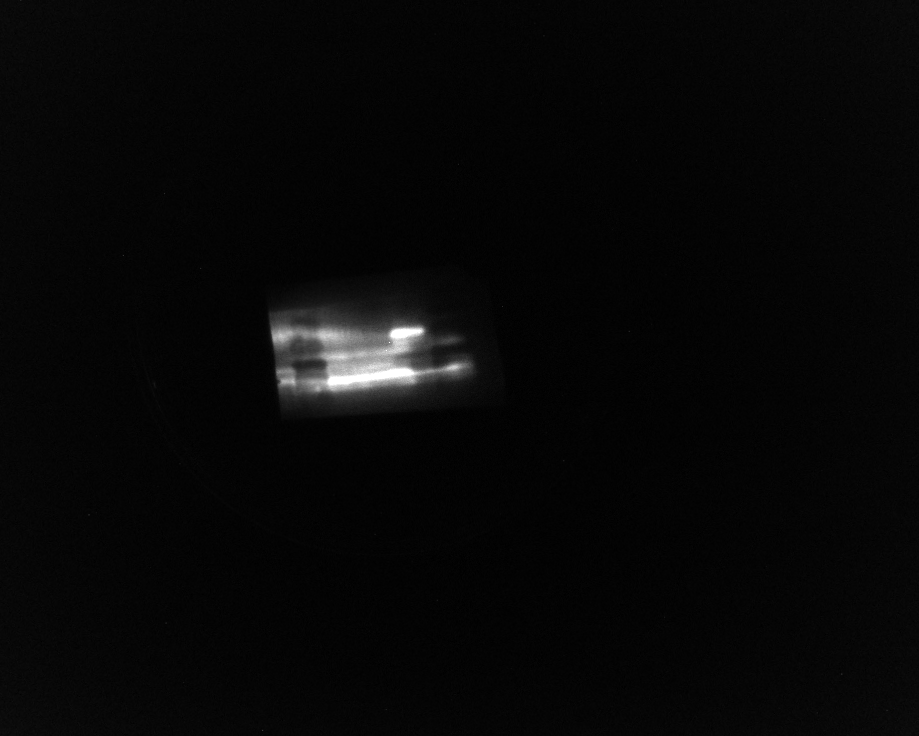

Supplement: Supplementary file 1 [file DataSheet1.zip › Fig6D ib fxr-fxr.tif]

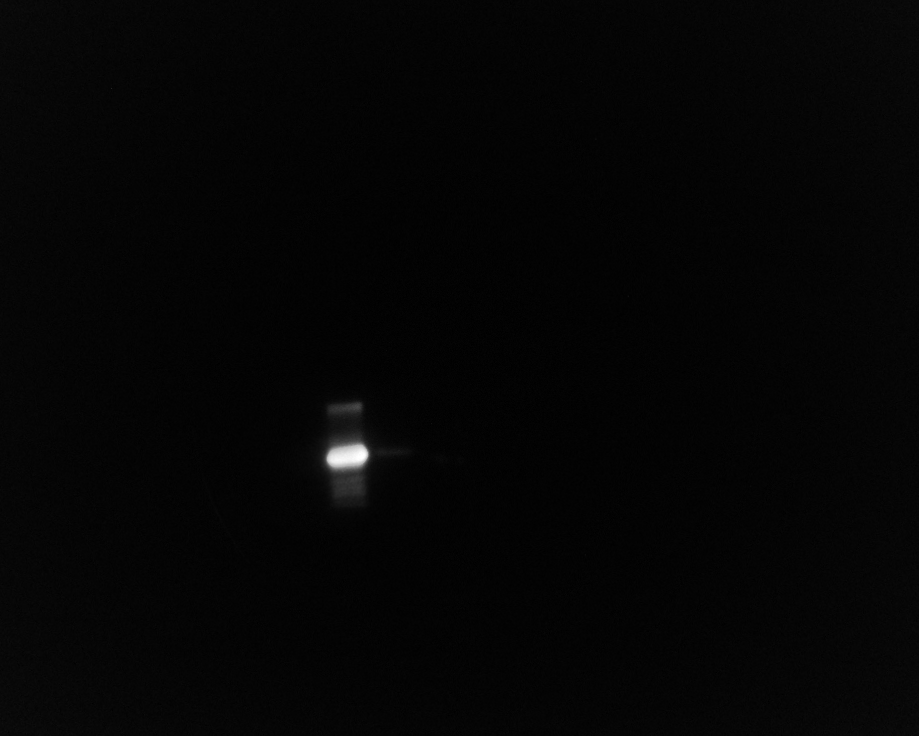

Supplement: Supplementary file 1 [file DataSheet1.zip › Fig6D ib fxr-RARa.tif]

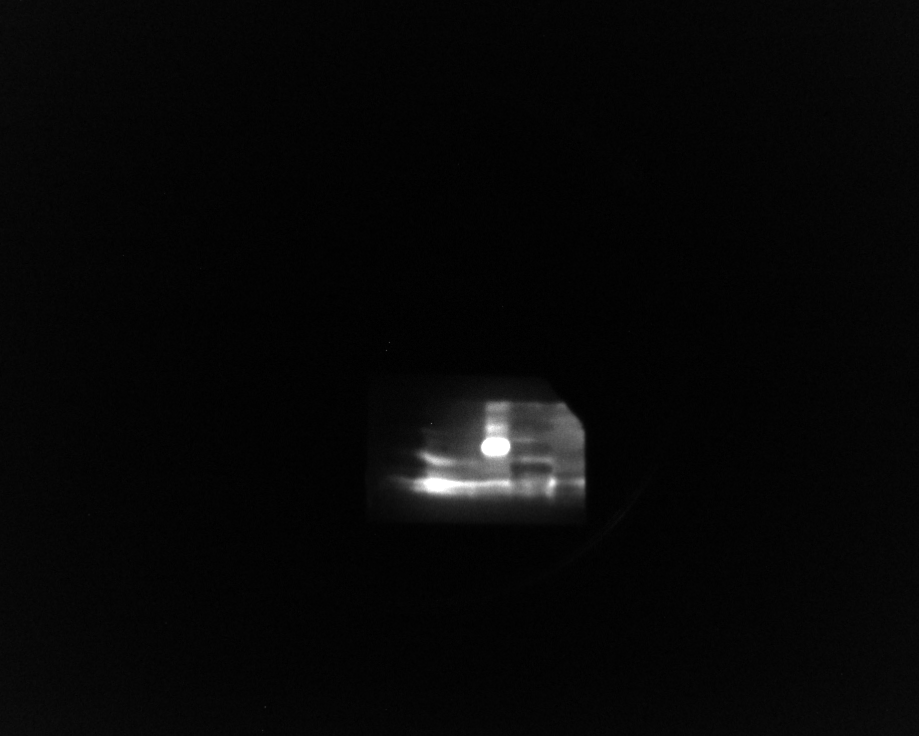

Supplement: Supplementary file 1 [file DataSheet1.zip › Fig6D ib RaRa-FXR.tif]

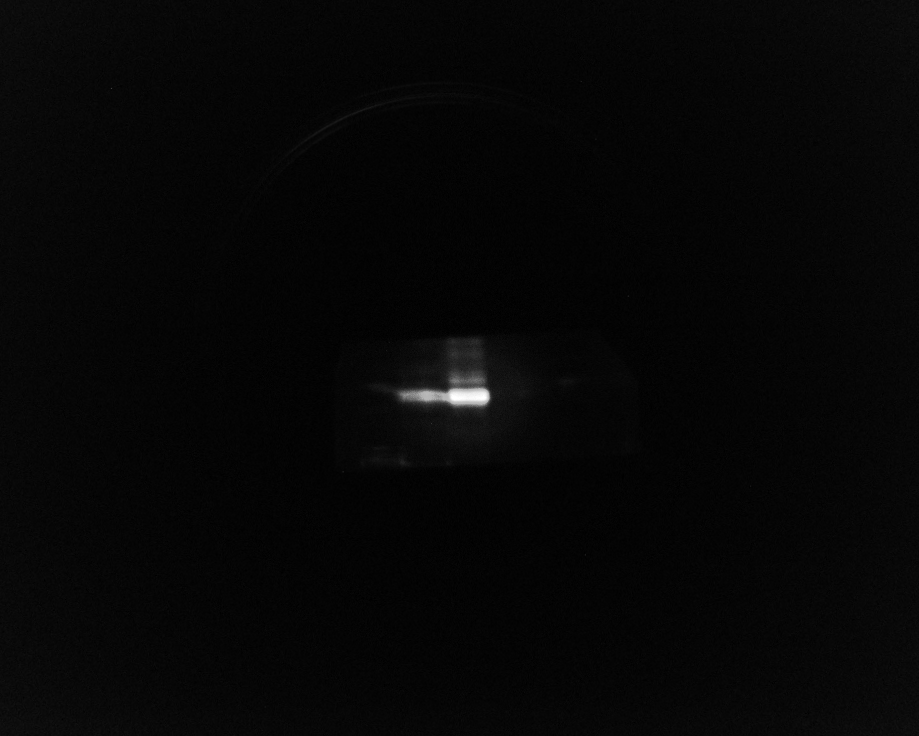

Supplement: Supplementary file 1 [file DataSheet1.zip › Fig6D ib RARa-RaRa.tif]

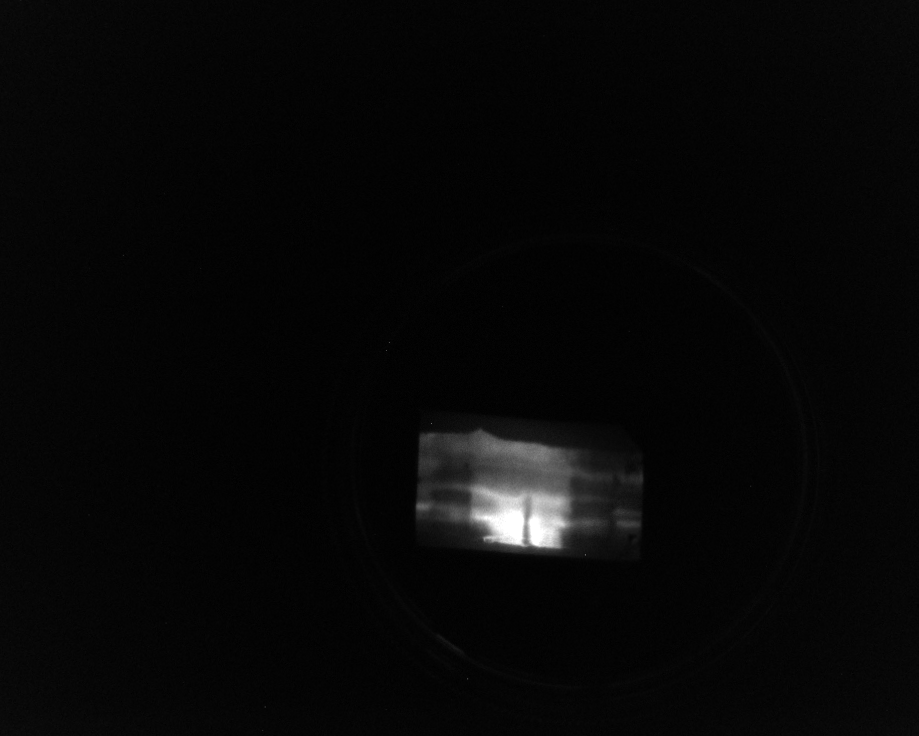

Supplement: Supplementary file 1 [file DataSheet1.zip › Fig6D input FXR.tif]

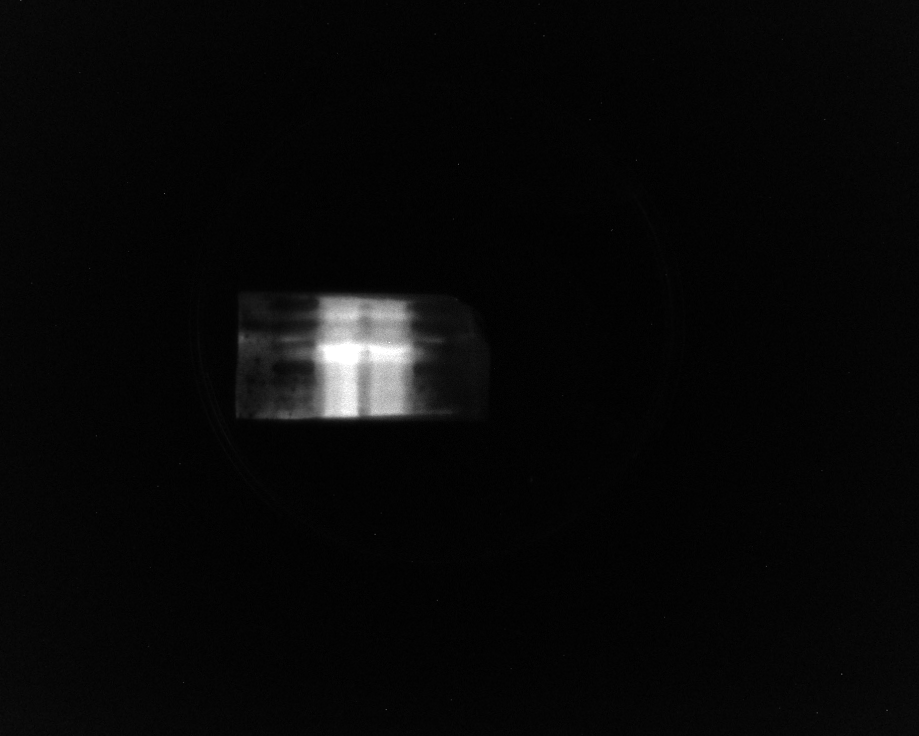

Supplement: Supplementary file 1 [file DataSheet1.zip › Fig6D input RARa.tif]

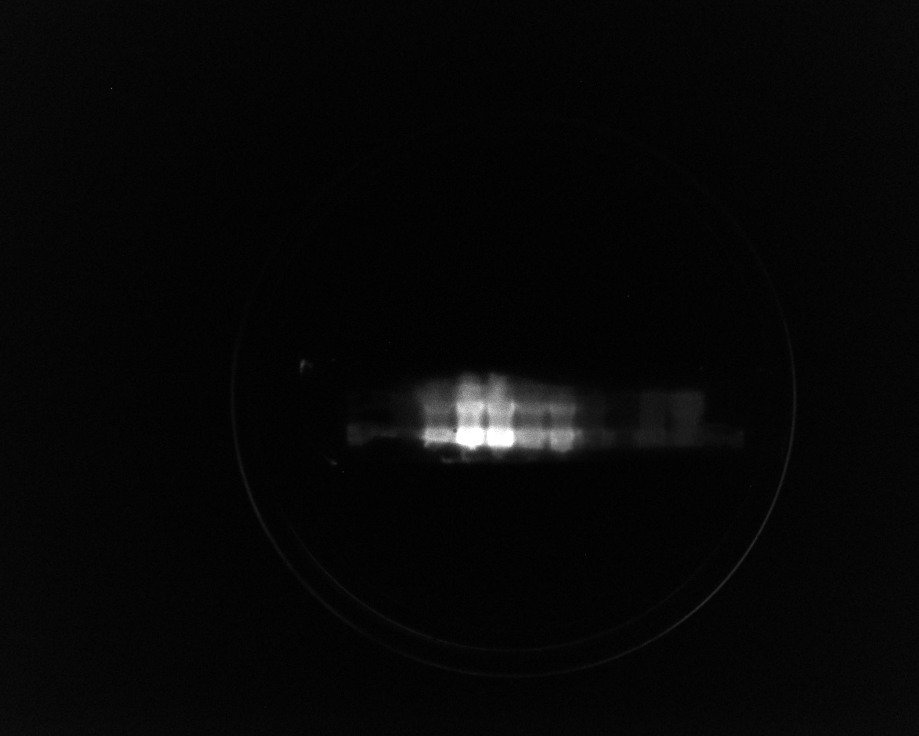

Supplement: Supplementary file 1 [file DataSheet1.zip › Fig6E FXR.tif]

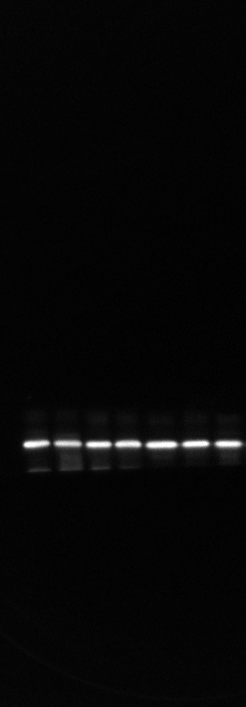

Supplement: Supplementary file 1 [file DataSheet1.zip › Fig6E gapdh.tif]

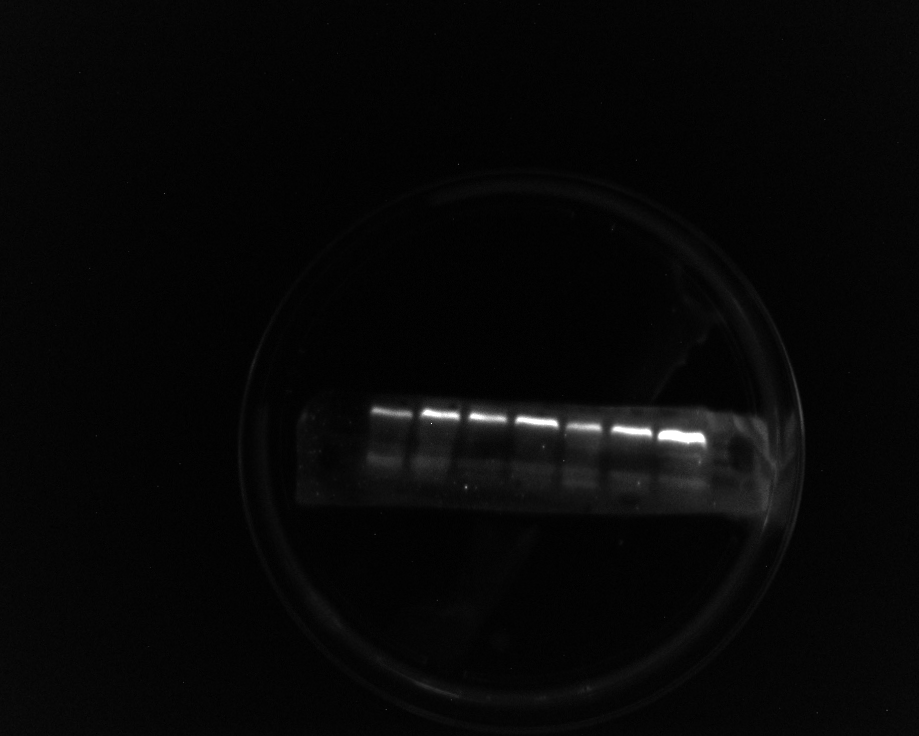

Supplement: Supplementary file 1 [file DataSheet1.zip › Fig6E RARa ser77.tif]

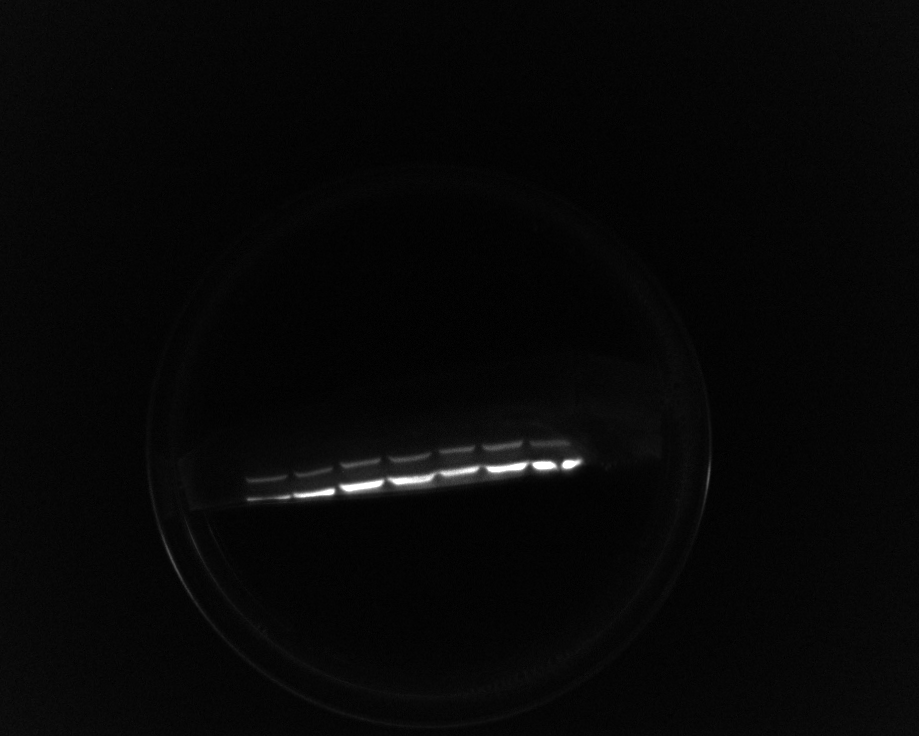

Supplement: Supplementary file 1 [file DataSheet1.zip › Fig6E RARa ser96.tif]

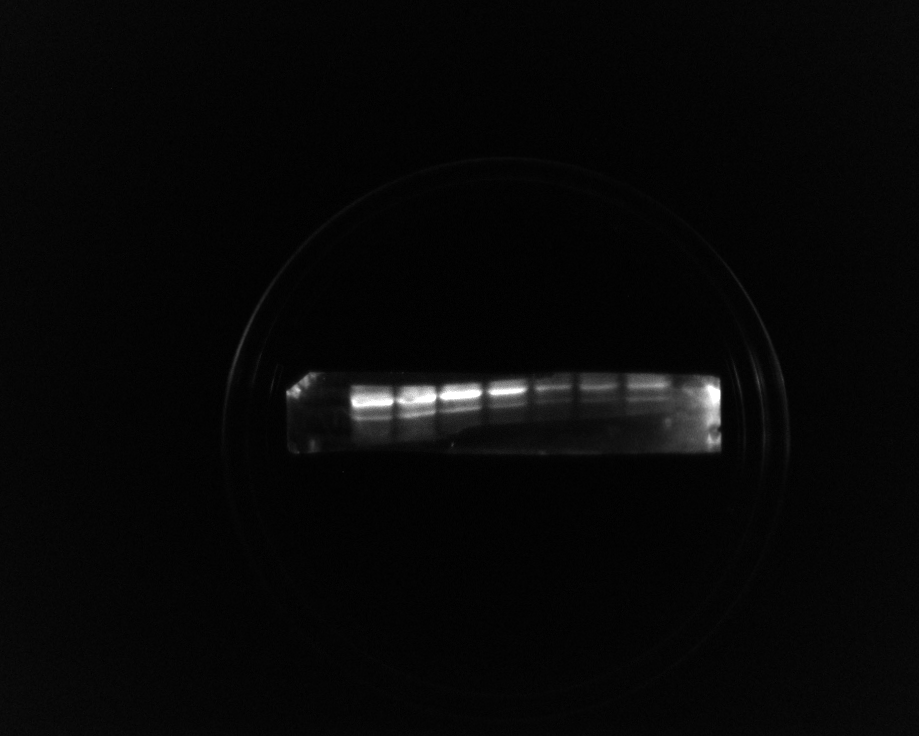

Supplement: Supplementary file 1 [file DataSheet1.zip › Fig6E rara.tif]

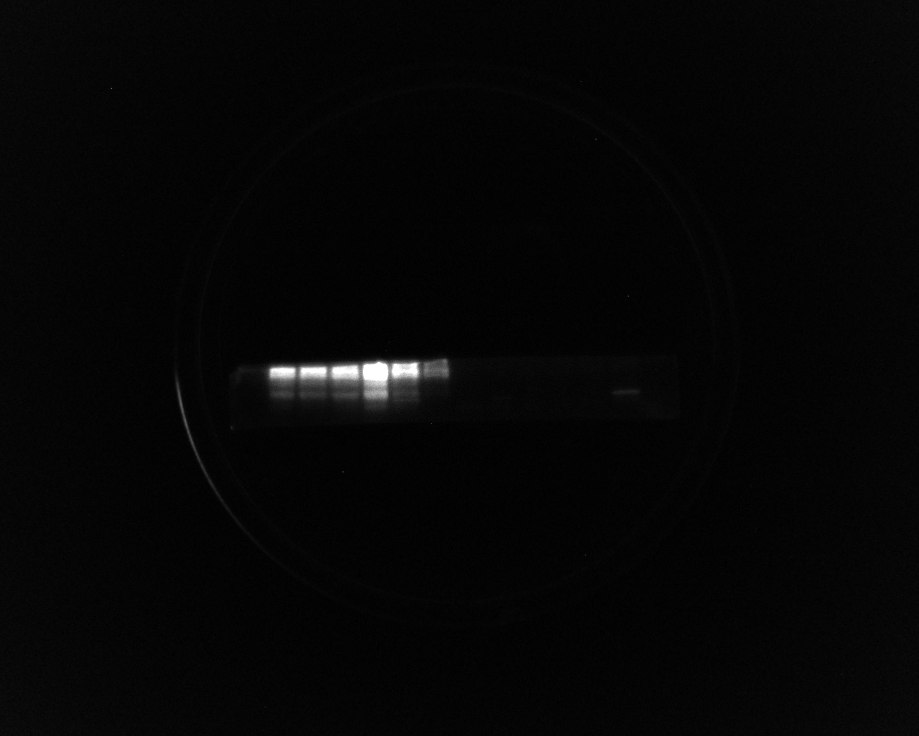

Supplement: Supplementary file 1 [file DataSheet1.zip › Fig6F FXR.tif]

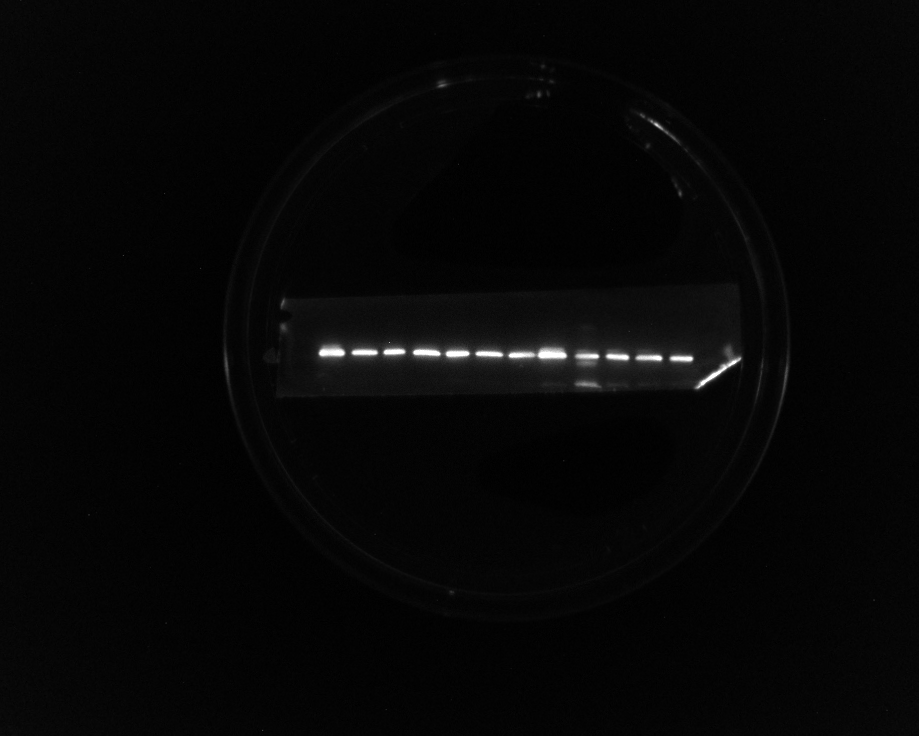

Supplement: Supplementary file 1 [file DataSheet1.zip › Fig6F GAPDH.tif]

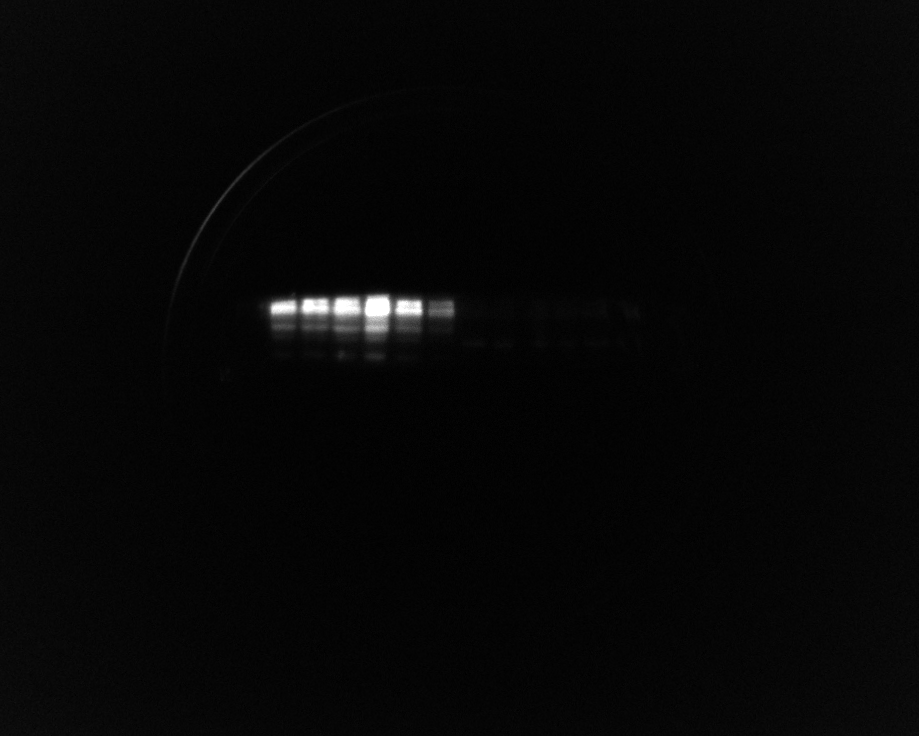

Supplement: Supplementary file 1 [file DataSheet1.zip › Fig6F RARA.tif]

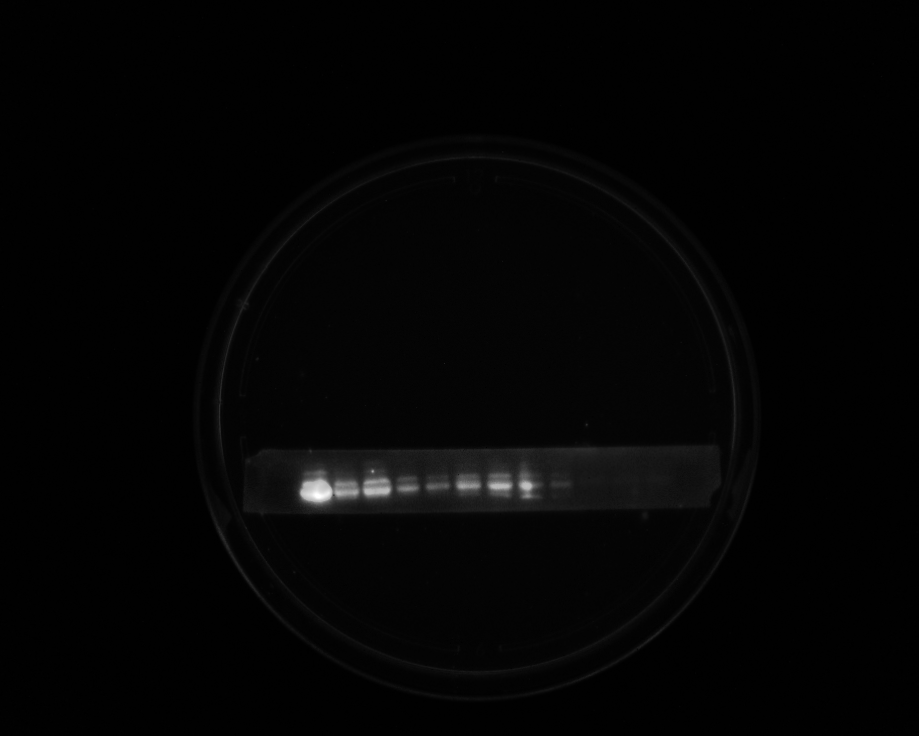

Supplement: Supplementary file 1 [file DataSheet1.zip › Fig6G FXR.tif]

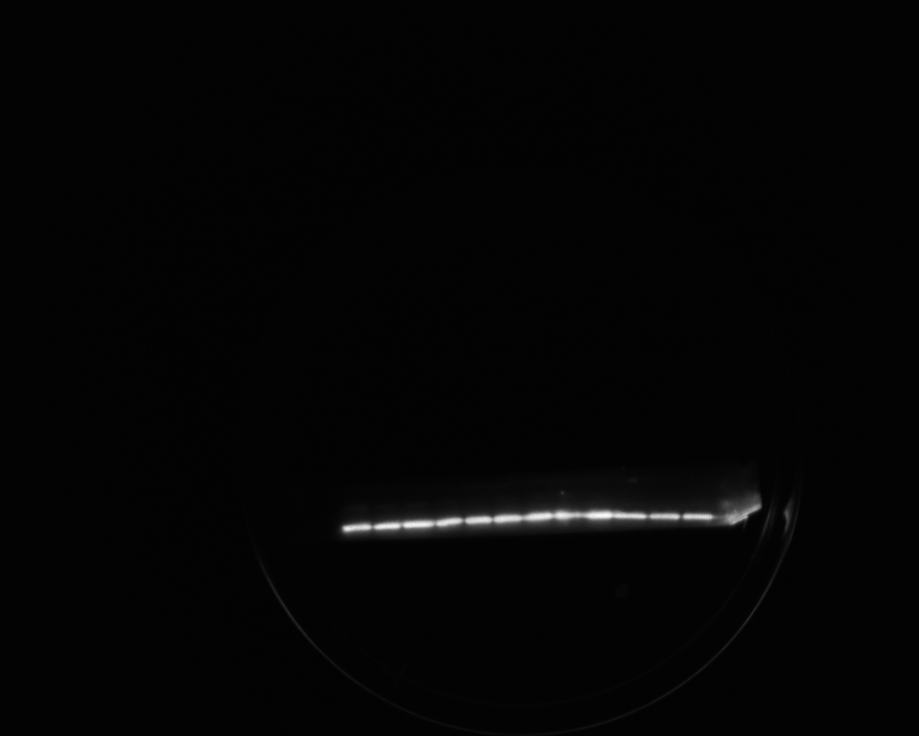

Supplement: Supplementary file 1 [file DataSheet1.zip › Fig6G GAPDH.tif]

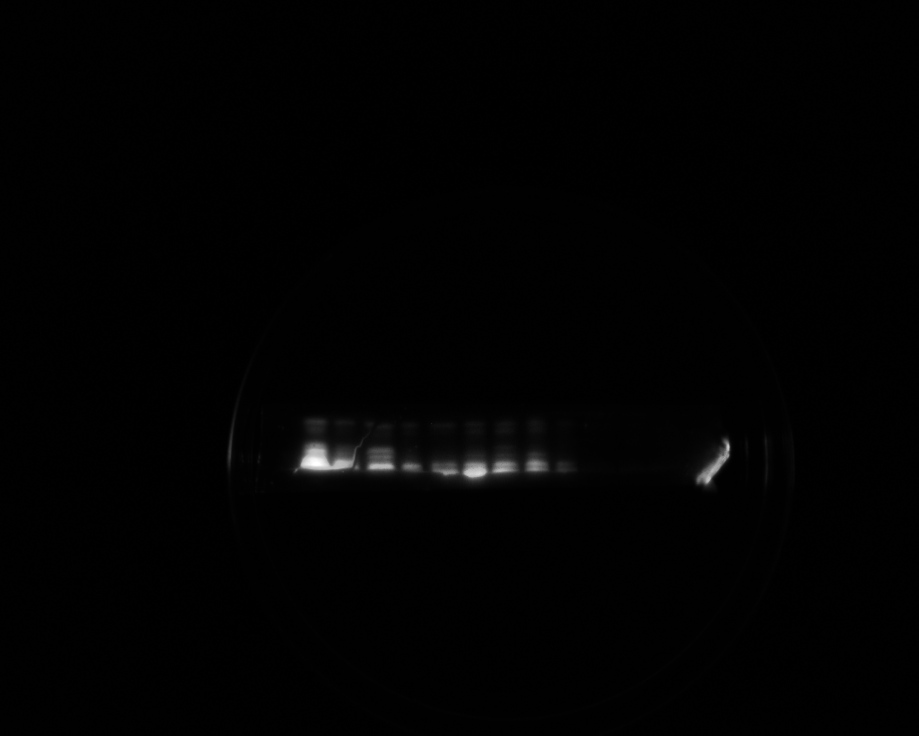

Supplement: Supplementary file 1 [file DataSheet1.zip › Fig6G RARA.tif]

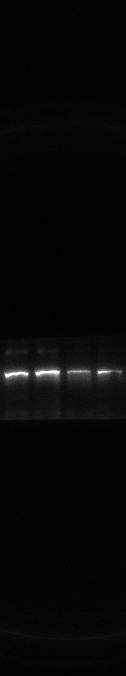

Supplement: Supplementary file 1 [file DataSheet1.zip › FigS4 fgfr2.tif]

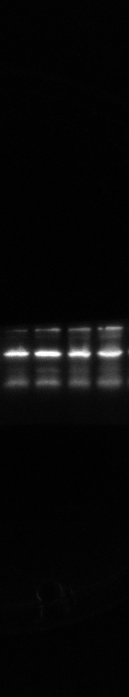

Supplement: Supplementary file 1 [file DataSheet1.zip › FigS4 gapdh.tif]

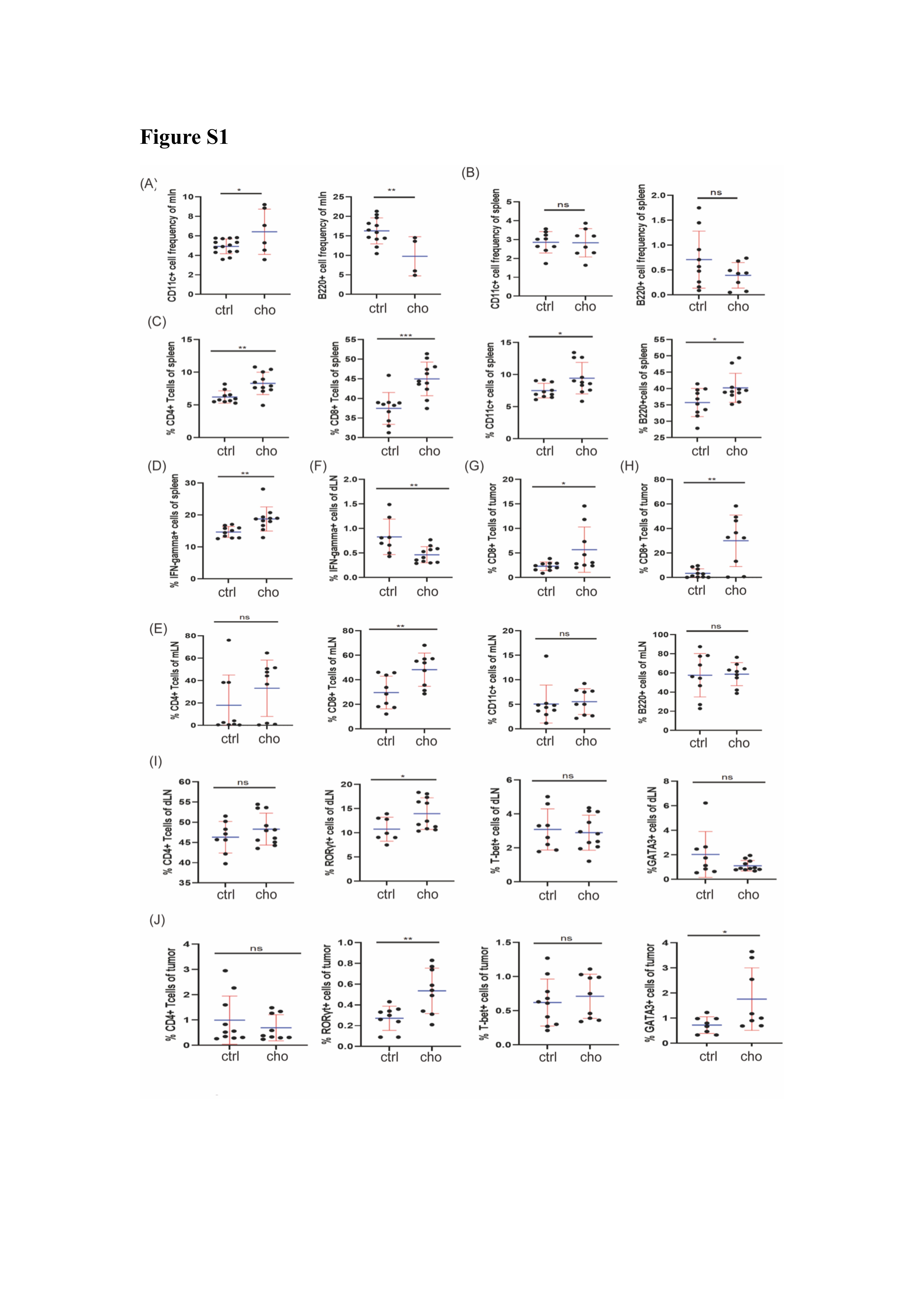

Supplement: Supplementary Figure 1 — Depleting bile acid stimulates an anti-tumor immune response. (A, B) The frequencies of CD11c+ and B220+ cells in the mesenteric lymph nodes (mLN) and spleen of the melanoma mouse model were analyzed by flow cytometry. (C, D) The frequencies of CD4+ T cells, CD8+ T cells, CD11c+ cells, B220+ cells, and IFN-γ+ cells in the spleens of 4T1 breast cancer mice were analyzed by flow cytometry. (E) CD4+ T cells, CD8+ T cells, CD11c+ cells, and B220+ cells frequency in mLN were determined by flow cytometry in the 4T1 breast cancer mouse model. (F) IFN-γ+ cell frequency in dLN was analyzed by flow cytometry in the 4T1 breast cancer mouse model. (G, H) The frequency of CD8+ T cells in the melanoma mouse model was determined by flow cytometry. (I, J) CD4+ T cells, RORγt+ cells, T-bet+ cells, and GATA3+ cells were quantified in the draining lymph node (dLN) and tumor by flow cytometry. Data are presented as mean ± SD; p-values were calculated using the two-tailed Student’s t-test. *p < 0.05, **p < 0.01, ***p < 0.001, ****p < 0.0001, n.s., not significant. [file Image1.jpeg]

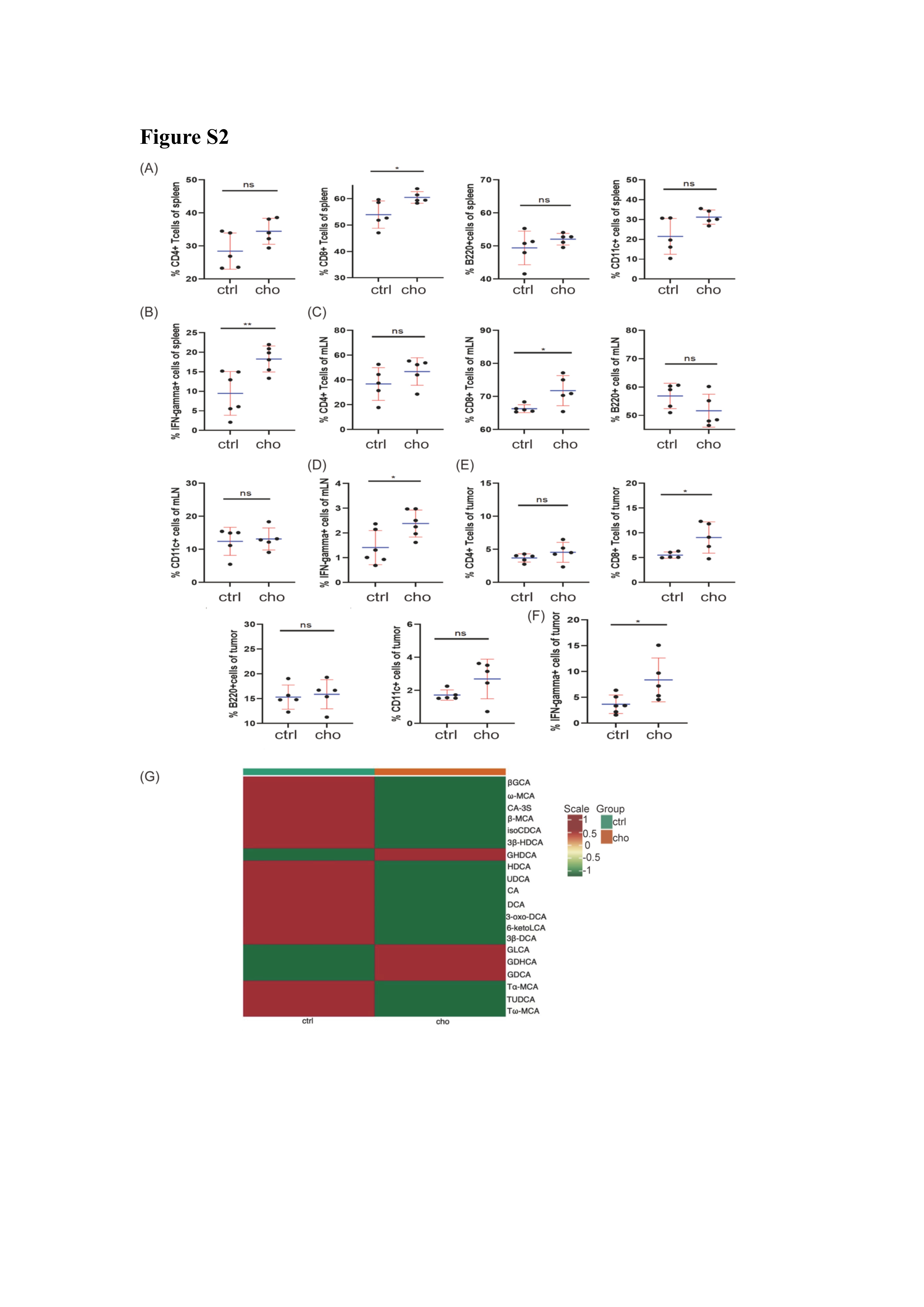

Supplement: Supplementary Figure 2 — The effect of depleting bile acid on immune cells and bile acid in the MMTV-PyMT mouse breast cancer model. (A) CD4+ T cells, CD8+ T cells, CD11c+ cells, and B220+ cells frequency in the spleen were analyzed by flow cytometry. (B) IFN-γ+ cell frequency in the spleen was analyzed by flow cytometry. (C) The frequency of CD4+ T cells, CD8+ T cells, CD11c+ cells, and B220+ cells in mLN was determined by flow cytometry. (D) IFN-γ+ cells frequency in mLN was analyzed by flow cytometry. (E) The frequency of CD4+ T cells, CD8+ T cells, CD11c+ cells, and B220+ cells in the tumor was examined by flow cytometry. (F) IFN-γ+ cells frequency in the tumor was analyzed by flow cytometry. (G) MMTV-PyMT mice were fed either a 2% cholestyramine diet or a regular diet. For about 20 days, bile acid profiles in the tumor were analyzed by UPLC-MS. The hierarchical clustering datasets were processed in R. Data are presented as mean ± SD; p-values were calculated using the two-tailed Student’s t-test. *p < 0.05, **p < 0.01, ***p < 0.001, ****p < 0.0001, n.s., not significant. [file Image2.jpeg]

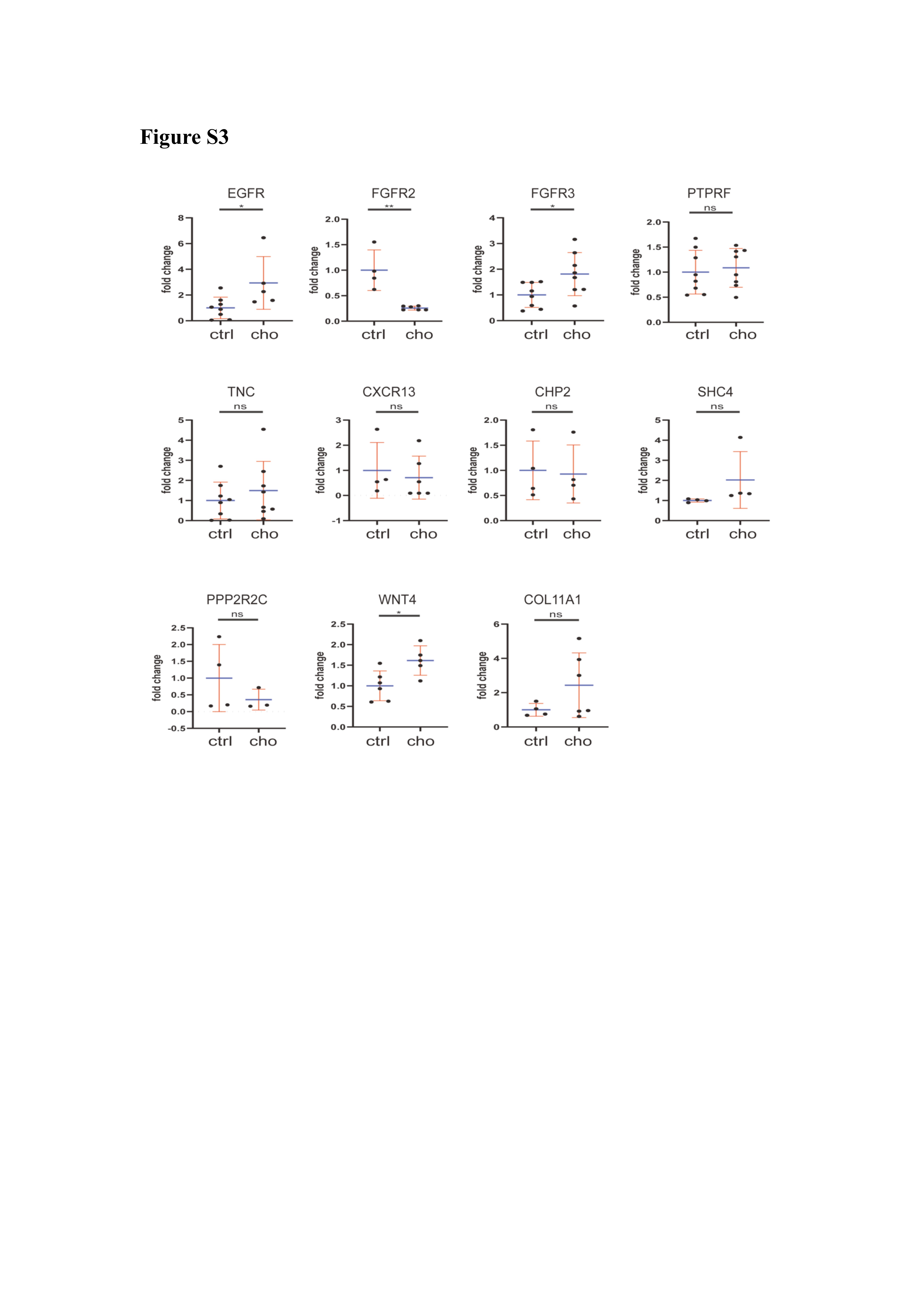

Supplement: Supplementary Figure 3 — Melanoma biomarker expression by RT-qPCR in a melanoma model. RT-qPCR was used to examine the expression levels of biomarker genes in the melanoma model. Data are presented as mean ± SD; p-values were calculated using the two-tailed Student’s t-test. *p < 0.05, **p < 0.01, ***p < 0.001, ****p < 0.0001, n.s., not significant. [file Image3.jpeg]

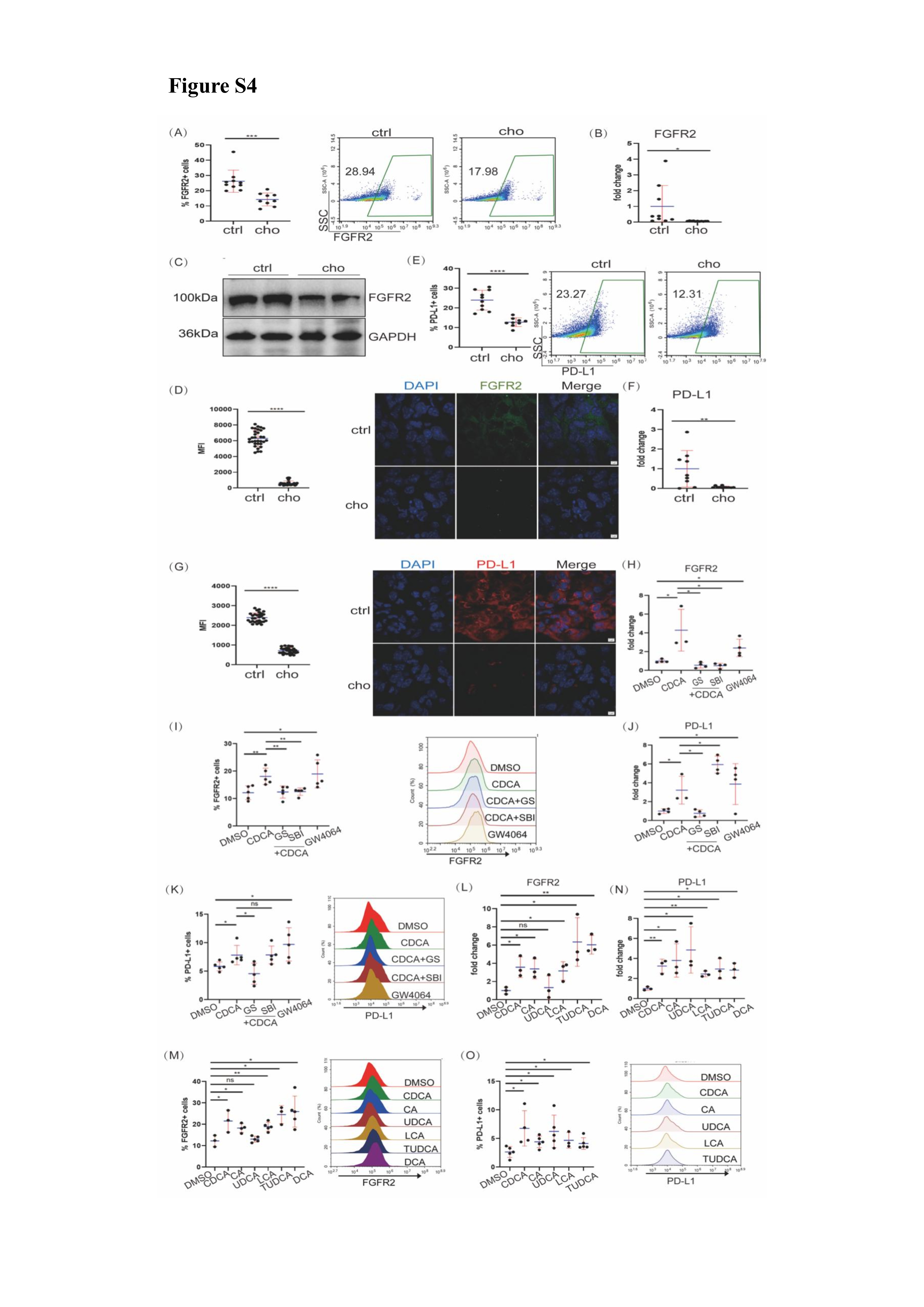

Supplement: Supplementary Figure 4 — Bile acids stimulate FGFR2 and PD-L1 expression in breast cancer cells. (A) FGFR2 expression level was analyzed by flow cytometry in 4T1 breast cancer. (B) RT-qPCR analyzed the FGFR2 mRNA expression level in 4T1 breast cancer. (C) FGFR2 expression level was determined by Western blot in 4T1 breast cancer. (D) Immunofluorescent analysis of FGFR2 in 4T1 breast cancer by 2% cholestyramine-fed mice. Breast cancer cells were stained with DAPI (blue) and FGFR2 (green) antibodies and analyzed by confocal imaging. The scale bars are 5μm. (E) PD-L1 expression level was determined by flow cytometry in 4T1 breast cancer. (F) PD-L1 mRNA expression level was determined by RT-qPCR in 4T1 breast cancer. (G) Immunofluorescent analysis of PD-L1 in 4T1 breast cancer by 2% cholestyramine-fed chow mice. Breast cancer cells were stained with DAPI (blue) and PD-L1 (red) antibodies and analyzed by confocal imaging. The scale bars are 5μm. (H) CDCA, CDCA+Guggulsterone, CDCA+SBI-115, and GW4064 stimulated MCF-7 cells, and RT-qPCR was used to examine FGFR2 mRNA expression levels. (I) CDCA, CDCA+Guggulsterone, CDCA+SBI-115, and GW4064 stimulated MCF-7 cells, and the FGFR2 expression level was examined by flow cytometry. (J) CDCA, CDCA+Guggulsterone, CDCA+SBI-115, and GW4064 stimulated MCF-7 cells, and RT-qPCR was used to examine PD-L1 mRNA expression levels. (K) CDCA, CDCA+Guggulsterone, CDCA+SBI-115, and GW4064 stimulated MCF-7 cells, and the PD-L1 expression level was examined by flow cytometry. (L) MCF-7 cells were stimulated by CDCA, CA, UDCA, LCA, TUDCA, and DCA, and RT-qPCR was used to analyze the FGFR2 mRNA expression level. (M) MCF-7 cells were incubated with CDCA, CA, UDCA, LCA, TUDCA, and DCA, and FGFR2-positive cells were analyzed by flow cytometry. (N) CDCA, CA, UDCA, LCA, TUDCA, and DCA stimulated MCF-7 cells, and RT-qPCR was used to analyze PD-L1 mRNA expression levels. (O) MCF-7 cells were incubated with CDCA, CA, UDCA, LCA, and TUDCA, and PD-L1-positive cells were analyzed by [file Image4.jpeg]

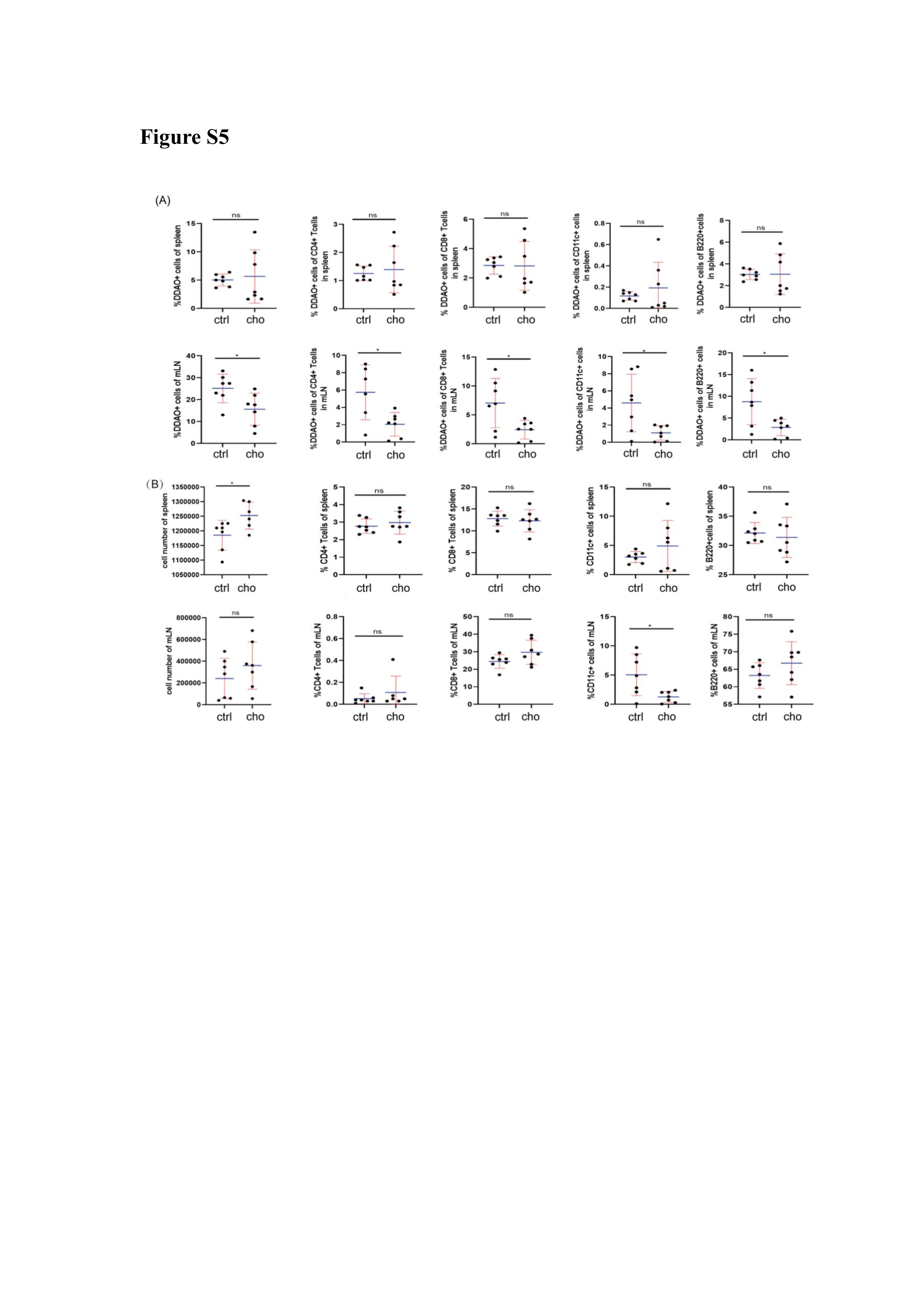

Supplement: Supplementary Figure 5 — The effect of cholestyramine treatment on immune cells. (A) RA reporter mice were treated with cholestyramine for 4 weeks. DDAO fluorescent signals of cells (Total, CD11c+, CD4+, CD8+, B220+) from the spleen and mLN were analyzed by flow cytometry. (B) Total cell number from spleen and mLN, CD11c+ cells, CD4+ T cells, CD8+ T cells, and B220+ cells frequency in spleen and mLN were determined by flow cytometry. Data are presented as mean ± SD; p-values were calculated using the two-tailed Student’s t-test. *p < 0.05, **p < 0.01, ***p < 0.001, ****p < 0.0001, n.s., not significant. [file Image5.jpeg]

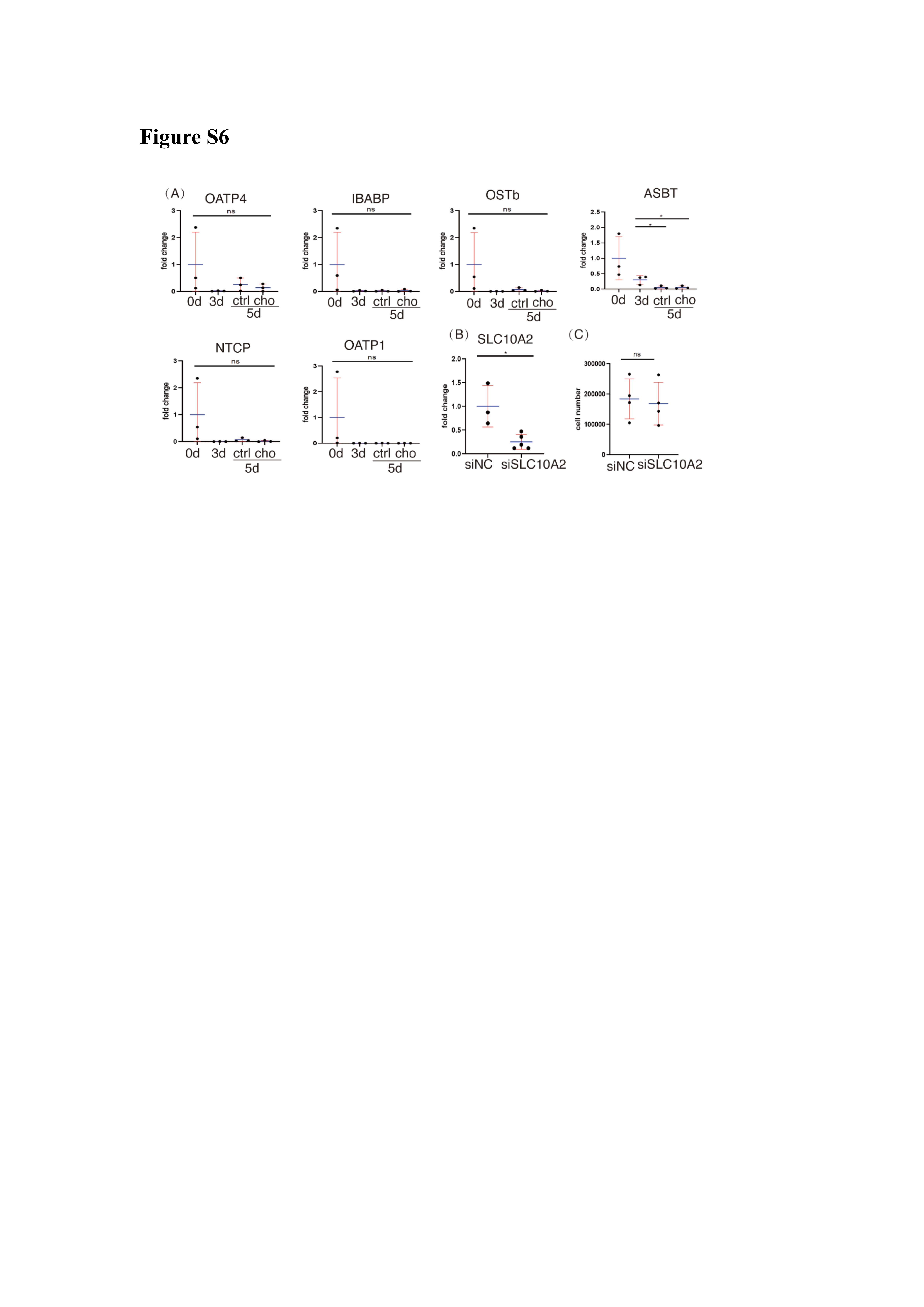

Supplement: Supplementary Figure 6 — Expression of bile acid transporter genes during tumor growth (A) RT-qPCR determined OATP4, IBABP, OSTb, SLC10A2, NTCP, and OATP1 mRNA in melanomas. (B) SLC10A2 mRNA expression after B16 cells were transfected with siSLC10A2 for 36 hours by qPCR. (C) B16 cells were transfected with siSLC10A2 for 36 hours to obtain cell numbers by FCM. Data are presented as mean ± SD; p-values were calculated using the two-tailed Student’s t-test. *p < 0.05, **p < 0.01, ***p < 0.001, ****p < 0.0001, n.s., not significant. [file Image6.jpeg]
